# The steroid hormone 20-hydroxyecdysone binds to dopamine receptor to repress lepidopteran insect feeding and promote pupation

**DOI:** 10.1371/journal.pgen.1008331

**Published:** 2019-08-14

**Authors:** Xin-Le Kang, Jun-Ying Zhang, Di Wang, Yu-Meng Zhao, Xiao-Lin Han, Jin-Xing Wang, Xiao-Fan Zhao

**Affiliations:** Shandong provincial key laboratory of animal cells and developmental biology, School of life science, Shandong University, Qingdao, China; University of Kentucky, UNITED STATES

## Abstract

Holometabolous insects stop feeding at the final larval instar stage and then undergo metamorphosis; however, the mechanism is unclear. In the present study, using the serious lepidopteran agricultural pest *Helicoverpa armigera* as a model, we revealed that 20-hydroxyecdysone (20E) binds to the dopamine receptor (DopEcR), a G protein-coupled receptor, to stop larval feeding and promote pupation. DopEcR was expressed in various tissues and its level increased during metamorphic molting under 20E regulation. The 20E titer was low during larval feeding stages and high during wandering stages. By contrast, the dopamine (DA) titer was high during larval feeding stages and low during the wandering stages. Injection of 20E or blocking dopamine receptors using the inhibitor flupentixol decreased larval food consumption and body weight. Knockdown of *DopEcR* repressed larval feeding, growth, and pupation. 20E, via DopEcR, promoted apoptosis; and DA, via DopEcR, induced cell proliferation. 20E opposed DA function by repressing DA-induced cell proliferation and AKT phosphorylation. 20E, via DopEcR, induced gene expression and a rapid increase in intracellular calcium ions and cAMP. 20E induced the interaction of DopEcR with G proteins αs and αq. 20E, via DopEcR, induced protein phosphorylation and binding of the EcRB1-USP1 transcription complex to the ecdysone response element. DopEcR could bind 20E inside the cell membrane or after being isolated from the cell membrane. Mutation of DopEcR decreased 20E binding levels and related cellular responses. 20E competed with DA to bind to DopEcR. The results of the present study suggested that 20E, via binding to DopEcR, arrests larval feeding and promotes pupation.

## Author summary

The steroid hormone 20-hydroxyecdysone (20E) represses insect larval feeding and promotes metamorphosis; however, the mechanism is unclear. The dopamine receptor plays important roles in animal motor function and reward-motivated behavior. Using the serious lepidopteran agricultural pest *Helicoverpa armigera* as a model, we revealed that 20E binds to DopEcR to block the dopamine pathway and initiates the 20E pathway. Dopamine (DA) binds to the dopamine receptor (DopEcR), a G protein-coupled receptor (GPCR), to regulate cell proliferation, larval feeding, and growth. However, 20E competes with DA to bind to DopEcR, which represses larval feeding and triggers the 20E-pathway, leading to metamorphosis. The results suggested that 20E, via binding to DopEcR, stops larval feeding and promotes pupation, which presented an example of the steroid hormone regulating dopamine receptor and behavior. Our study showed that GPCRs can bind 20E and function as 20E cell membrane receptors.

## Introduction

The post-embryo development of holometabolous insects involves larval, pupal, and adult stages. The transformation from the final instar larva to the adult is called metamorphosis. During metamorphosis, the larvae stop eating, start wandering, and finally become quiescent before pupating. The insect molting hormone 20-hydroxyecdysone (20E) promotes metamorphosis by upregulating 20E-pathway gene expression [1] and by counteraction with the juvenile hormone [2] and insulin [3]. However, the regulatory mechanism by which larvae stop feeding is unclear.

20E initiates gene expression by binding to its nuclear receptor ecdysteroid hormone receptor B1 (EcRB1), which forms a transcription complex with ultraspiracle protein (USP1) and binds to the ecdysone response element (EcRE) [4]. However, as the mammal estrogen transmits signal via cell membrane receptor [5], 20E also induces signaling via G protein-coupled receptors (GPCRs). In *Bombyx mori*, 20E, via an unidentified GPCR, increases the intracellular Ca^2+^ level in the anterior silk gland [6] and activates the protein kinase C (PKC) pathway [7]. In *Helicoverpa armigera*, ErGPCR-1 [8] and ErGPCR-2 [9] transmit 20E signals in the cell membrane. 20E, via GPCR, Phospholipase C (PLC), and calcium-signaling pathways, regulates protein phosphorylation, including that of USP1 [10], cyclin dependent kinase 10 (CDK10) [11], and catalytic domain of protein kinase A (PKAC1) [12] to form the 20E transcription complex EcRB1/USP1 and promote gene expression during insect metamorphosis [10, 13].

GPCRs play fundamental roles in mediating various cellular responses from the extracellular environment, including ions, light, amines, odorants, lipids, peptides, amino acids, and nucleotides [14]. However, whether GPCRs are the cell membrane receptors for 20E or other steroid hormones remains controversial because a lack of direct evidence of GPCRs binding to steroid hormones. For example, human GPER (GPR30) [15] binding to Alexa 633-labeled estrogen was assayed using the COS7 cells that overexpress GPR30 [5]. The *Drosophila* dopamine receptor (DmDopEcR) binding of the 20E analog tritium-labeled ponasterone A ([^3^H]Pon A), was assayed using the cell membranes of Sf9 cells that overexpress DmDopEcR [16]. To date, there is no direct evidence to show that an isolated GPCR can bind a steroid hormone *in vitro*.

Dopamine receptors are typical GPCRs that localize to the cell membrane [17]. In vertebrates, dopamine receptors are mainly expressed in the central nervous system (CNS), and are distributed in non-CNS tissues, such as in epicardium of the heart [18] and the nephron in the kidney [19]. Dopamine receptors are implicated in many neurological processes, including motivation, pleasure, cognition, memory, learning, and fine motor control, as well as modulation of neuroendocrine signaling [20]. Dopamine binds to its receptors to regulate important reward-motivated behaviors, including eating [21] and reward-induced eating behavior in humans [22]. The loss of dopamine neurons results in Parkinson’s disease, whereas hyperactive dopaminergic signaling might be a major factor in the positive symptoms of schizophrenia [23]. However, the regulators of dopamine receptors are not fully understood.

In insects, injection of dopamine initiates gregarious behavior of *Locusta* [24]. Dopamine receptors function to modulate phase change in the brain of *Locusta*, with dopamine receptor-1 induces the gregariousness and dopamine receptor-2 mediates the solitariness [25]. DmDopEcR is necessary for L-dopa-increased sugar sensitivity [26]. Reduced function of DopEcR in *Drosophila* mushroom bodies (MB) resulted in decreased-locomotor activity [27]. DmDopEcR requires ecdysone and dopamine as ligands to regulate the perception of sex pheromones [28, 29]. DmDopEcR bound either dopamine (DA) or [^3^H]Pon A when DmDopEcR was overexpressed in Sf9 cells. 20E can compete with [^3^H]Pon A to bind to DmDopEcR; therefore, DmDopEcR is considered as a cell membrane receptor of 20E [16]. However, the functions of dopamine receptors in insects are poorly understood.

In the present study, we showed that DopEcR plays dual functions in insect feeding and pupation. DA, via DopEcR, regulates larval feeding and growth by promoting cell proliferation. 20E competes with DA to bind to DopEcR and uses DopEcR as one of its plasma membrane receptors to transmit the 20E signal to promote insect metamorphosis. DopEcR could bind 20E in the cell membrane and *in vitro* after it was isolated from the cell membrane. This study presents evidence that GPCRs function as cell membrane receptors of steroid hormones, and the interaction between the endocrine system and the nervous system.

## Results

### DopEcR is highly expressed in the brain under 20E regulation

**Fig 1:**
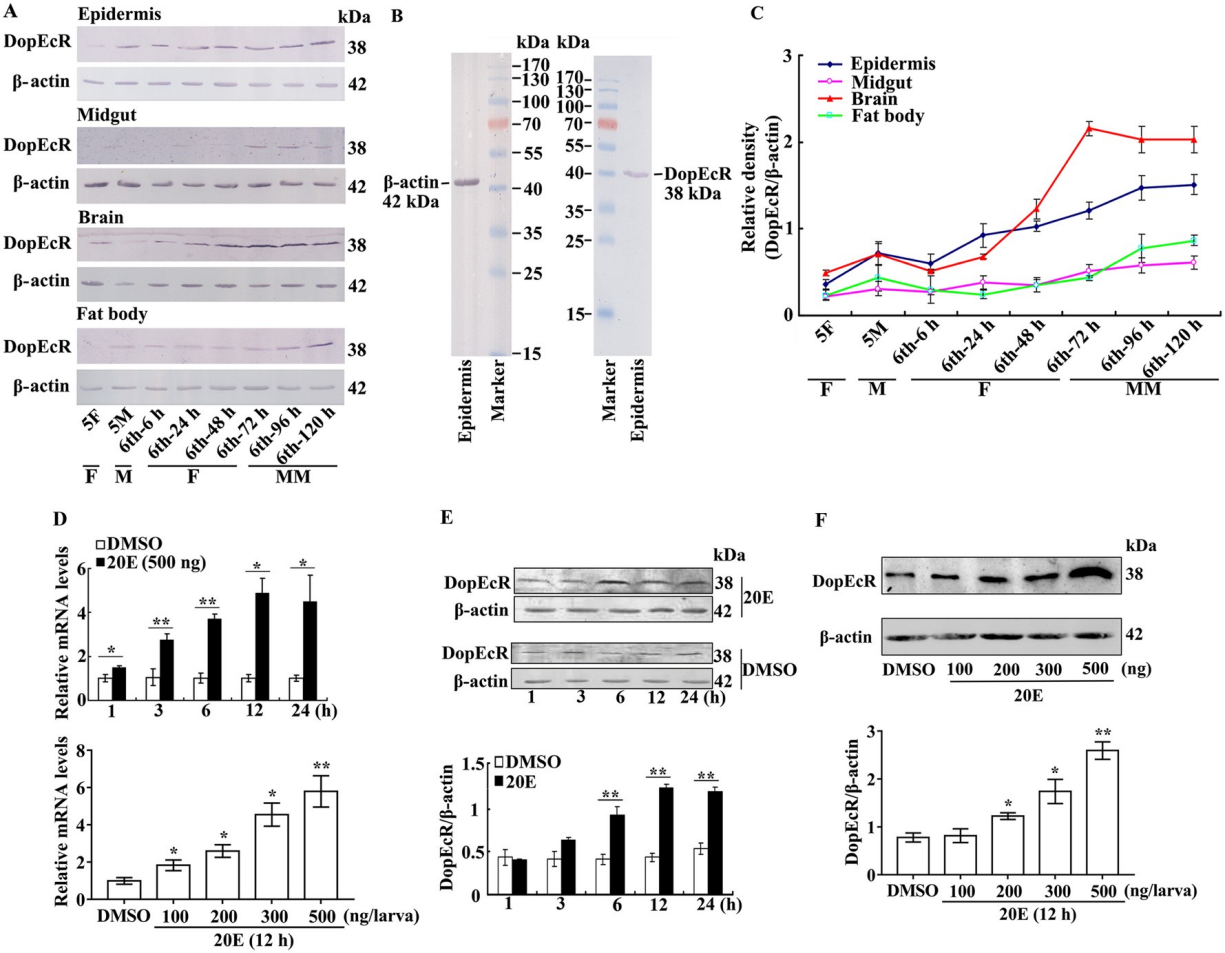
Expression profiles of DopEcR by western blot analysis and 20E upregulates DopEcR expression. **A**. Expression profiles of DopEcR detected by anti-DopEcR antibody as the method description. β-actin as control. **B**. Specificity analysis of the antibodies against DopEcR and β-actin by western blotting with epidermis protein of 6th-72 h larvae. SDS-PAGE gel in western blot is 12.5%. **C**. Quantification of the data in A by ImageJ. Error bars indicate the mean ± standard deviations (SD) of three times repetition. **D**. qRT-PCR analysis of 20E regulation on *DopEcR* mRNA in larval head by time and dose. Equal volume of diluted DMSO was injected as control. The relative mRNA level was counted by 2^–ΔΔCT^. **E**. and **F**. Western blot analysis of 20E regulation on DopEcR in larvae head as the treatment in D. SDS-PAGE gel in western blot is 12.5%. β-actin was detected as control. Statistical analyses of C and D by Student’s *t* test and ImageJ. Error bars showed the mean ± SD of three times repetition. Asterisks manifest significant differences by Student’s *t* test (*p < 0.05; **p < 0.01).

To study the function of DopEcR in *H*. *armigera*, the developmental expression profiles of DopEcR in tissues were examined. High levels of DopEcR were detected in the brain, which increased during metamorphic molting from the sixth instar (48 h to 120 h). In addition, DopEcR was detected in the epidermis, midgut, and fat body, and increased during metamorphic molting (Fig 1A–1C), which suggested that DopEcR plays roles in the brain and other tissues.

To examine 20E-induced regulation on *DopEcR* expression, sixth instar 6 h larvae were injected with 20E. The mRNA levels of *DopEcR* in the head were upregulated by 20E induction in a time and dose-dependent manner, as assessed using quantitative real-time reverse transcription PCR (qRT-PCR) analysis (Fig 1D). Western blot analysis also detected significant upregulation of DopEcR expression in the larval head in response to 20E induction in a time and dose-dependent manner (Fig 1E and 1F). These results showed that 20E upregulates the expression of DopEcR.

### 20E represses larval food feeding and DA is involved in larval feeding

**Fig 2:**
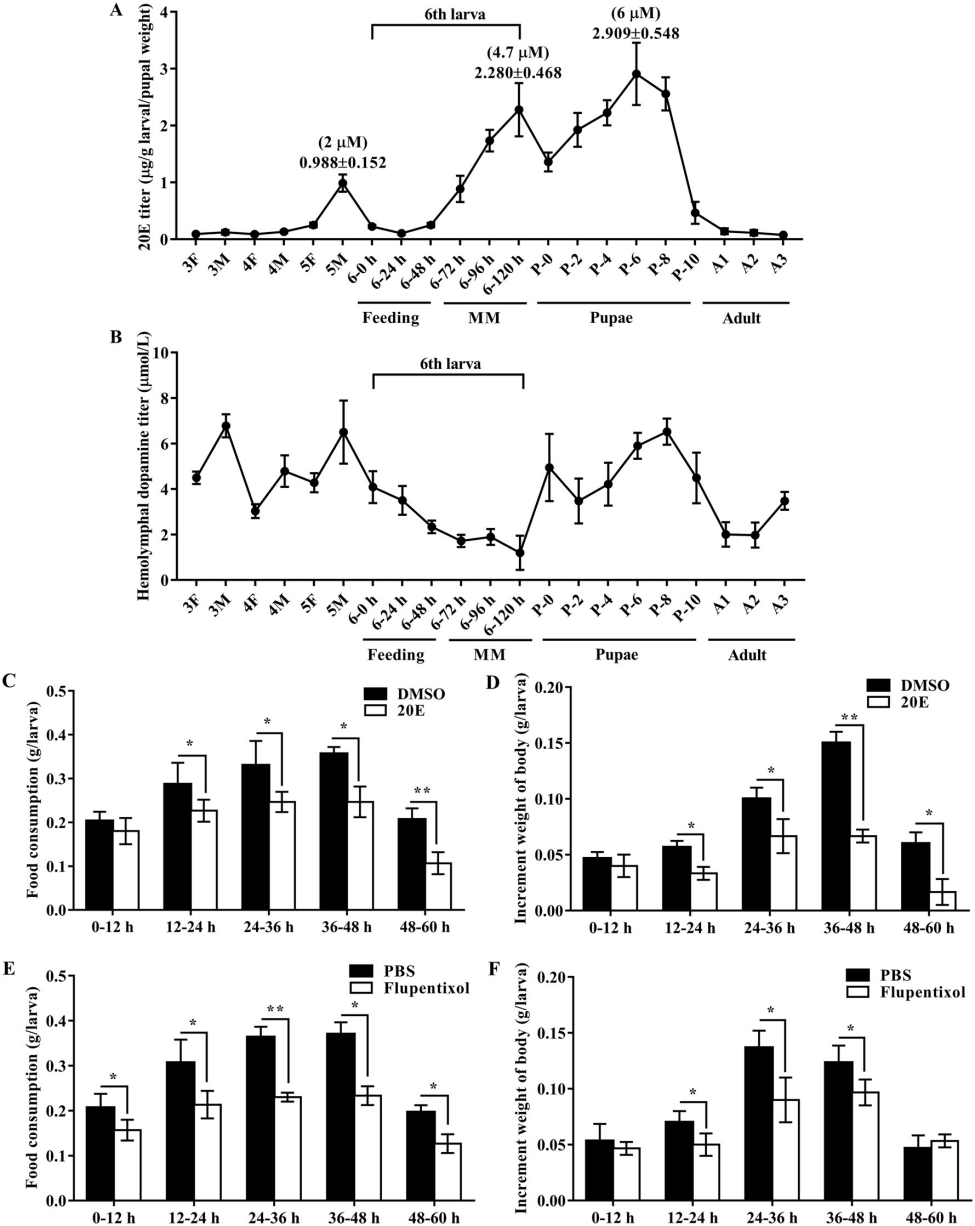
The 20E titer and dopamine titer in *H*. *armigera* and 20E repressed larval feeding and promoted pupation. **A**. 20E titer of the whole body from 3rd instar larvae to adult 3 day (A3). **B**. Hemolymphal dopamine titer from 3rd instar larvae to adult 3 day (A3). 3F: third instar feeding larvae; 3M: third instar molting larvae; 4F: fourth instar feeding larvae; 4M: fourth instar molting larvae. 5F: fifth instar feeding larvae; 5M: fifth instar molting larvae; 6–0 h, 6–24 h, 6–48 h, 6–72 h, 6–96 h, and 6–120 h: sixth instar larvae from 0 h to 120 h; P0, P2, P4, P6, P8, P10: zero to 10-day-old pupae. A1-A3: adult day 1 to day 3 from female adult. **MM**: metamorphic molting. **C**. The food consumption was quantitated as the amount of diet eaten at 0–12, 12–24, 24–36, 36–48 and 48–60 h after 500 ng 20E injection. DMSO was used as control. The amount of the food consumption was weighted for the quantity of feeding. **D**. The increment weight of body was quantitated at 0–12, 12–24, 24–36, 36–48 and 48–60 h after 500 ng 20E injection. DMSO was used as control. **E**. The food consumption was quantitated as the amount of diet eaten at 0–12, 12–24, 24–36, 36–48 and 48–60 h after flupentixol injection. PBS was used as control. The amount of the food consumption was weighted for the quantity of feeding. **F**. The increment weight of body was quantitated at 0–12, 12–24, 24–36, 36–48 and 48–60 h after flupentixol injection. PBS was used as control. Error bars show the mean ± SD of three biological repeats. Significant differences were calculated by Student’s *t* test (*p < 0.05; **p < 0.01).

We analyzed the 20E titer in whole body and the DA titer in hemolymph from 3rd instar larvae to adults to analyze the roles of 20E and DA in larval development. The results showed that the 20E titer was low in earlier instar larvae. However, the 20E titer increased markedly from 6th instar 72 h to the late pupal stage. The highest titer was 6 μM (2.9 μg/g larval body weight), which was detected in the pupal stage (Fig 2A). By contrast, the DA titer was high in earlier instar larvae, and declined from the 6th instar at 0 h to 120 h (Fig 2B), suggesting opposite functions of 20E and DA in metamorphosis and larval feeding and growth.

To examine the influence of 20E on larval food consumption, 500 ng of 20E was directly injected into each *H*. *armigera* larva at the sixth instar 6 h stage and food consumption and increment weight of body were monitored. 20E injection decreased food consumption from 21% to 30% from 24 h to 60 h (Fig 2C). The average body weight increased at a significantly slower rate by 20E injection compared with that in larvae treated with dimethyl sulfoxide (DMSO) (Fig 2D). These data suggested that the 20E repressed larval food consumption, resulting in weight reduction. In addition, we injected the inhibitor of dopamine receptor, flupentixol [16], into the larva at sixth instar 6 h to reach the final concentration of 50 μM. The average feeding quantity and the increase in body weight decreased compared with those in the PBS-treated controls (Fig 2E and 2F), suggesting that DopEcR functions in larval feeding and growth.

### Knockdown of *DopEcR* decreases feeding and delayed pupation time

**Fig 3:**
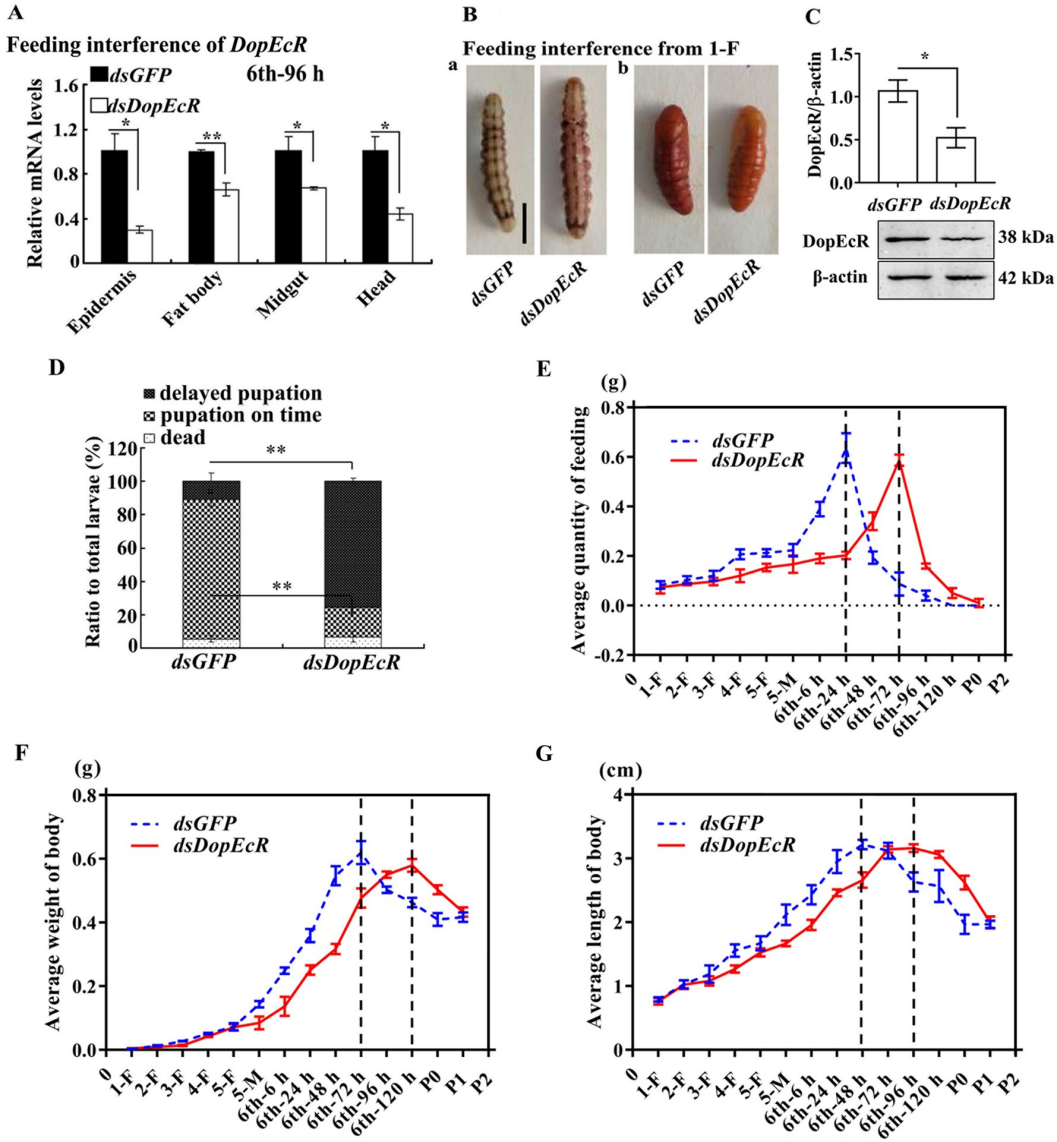
Silencing *DopEcR* by feeding dsRNA from first instar to 6th instar larvae repressed growth, feeding and pupation. **A**. The mRNA expression levels of *DopEcR* in tissues of 6th-96 h larvae were quantified by qRT-PCR after *DopEcR* knockdown. **B**. Phenotypes after feeding dsRNA. **a**. Phenotypes of larvae, with larger body size after feeding *dsDopEcR*. **b**. Phenotypes of pupae. The bar represents 1 cm. **C**. Western blotting showing the knockdown efficiency by dsRNA feeding (12.5% SDS gel with β-actin used as a control). ImageJ software was used to transform the image data. **D**. Ratio of Phenotypes. The data were calculated from 30 larvae × 3 experiments. **E. F**. and **G**. The average quantity of feeding, body weight and body length of insect from first instar larvae (1-F) to pupae (P-2), analyzed individually with 30 insects. All data were performed in triplicate. The bars indicate the mean ± SD. (*p < 0.05; **p < 0.01).

To determine the role of DopEcR in larval feeding and development, we knocked down *DopEcR* by feeding first-instar larvae with *Escherichia coli* (HT115) that expressed *dsGFP* or *dsDopEcR*, respectively. *DopEcR* was significantly knocked down in the epidermis, midgut, fat body, and head in larvae feed with feeding *dsDopEcR*-expressing *E*. *coli* (Fig 3A). The death rate in the *dsDopEcR* group and *dsGFP* group showed no significant difference; however, the time to shorten the larval body was delayed (the larval body was shortened at the normal prepupal stage) (Fig 3B). Western blotting demonstrated the efficacy of RNAi (Fig 3C). 75% of the larvae showed delayed pupation for approximately 44 h (Fig 3D). In addition, larval feeding was repressed and the highest consumption of food was postponed by 48 h (Fig 3E). The average body weight and body length were also decreased and postponed for 48 h (Fig 3F and 3G). The time to reach maximum body weight and body length was delayed for about 48 h in the *dsDopEcR*-treated animals. There was no significant difference in the final body weight and body length between the larvae treated with *dsDopEcR* and those treated with *dsGFP*. These results suggested that DopEcR is necessary either for larval feeding or pupation.

**Fig 4:**
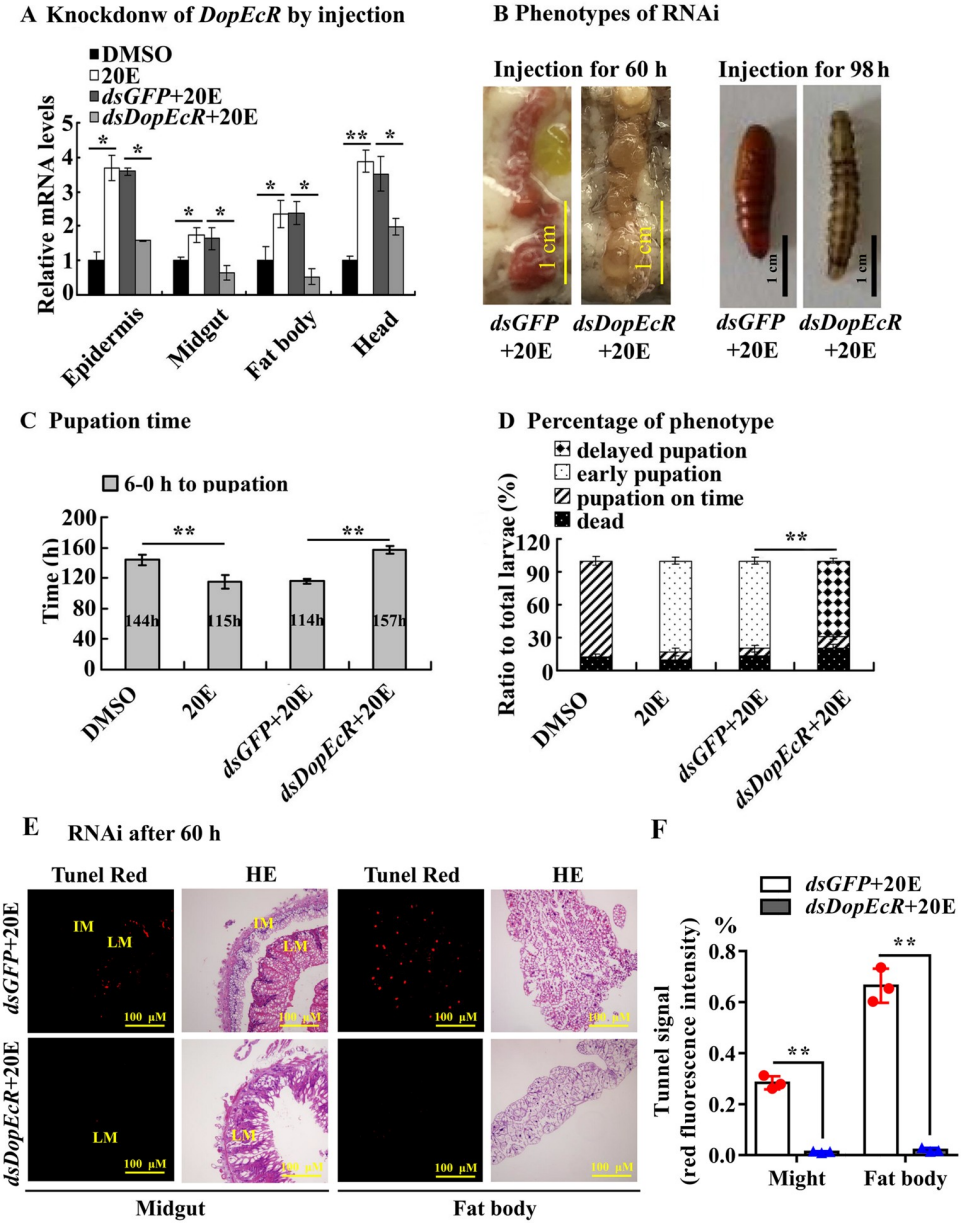
*DopEcR* silence by dsRNA injection delayed pupation and tissue remodeling. **A**. The mRNA expression levels of *DopEcR* in tissues of 6th-72 h larvae were quantified by qRT-PCR after *DopEcR* knockdown (sixth instar 6 h larvae for the first dsRNA injection, thrice at a 24 h interval, 500 ng dsRNA/larva). 20E (500 ng/larva). DMSO was a solvent control. **B**. Phenotypes after *DopEcR* knockdown as the experiments in A. Phenotypes were obtained at 98 h after first *dsDopEcR* injection. Scale bar = 1 cm. **C** and **D**. Statistical analysis of pupation time from sixth instar 6 h larvae developing to pupae and the percentage of different phenotypes. **E**. Tunel and HE-stained midgut and fat body after knockdown of *DopEcR*, observed at 60 h after the first dsRNA injection. LM: larval midgut; IM: imaginal midgut. Tunel Red fluorescence indicates apoptotic signal. HE staining showing the morphology of the midgut and fat body. The yellow bars represent 100 μM. **F**. The red fluorescence intensity (tunnel signal) were counted by ImageJ and represented with mean ± SD. Tunnel signal was calculated with red area (% of the tissue). Error bars show the mean ± SD of three biological repeats. Significant differences were calculated by Student’s *t* test (*p < 0.05; **p < 0.01).

To confirm the function of DopEcR in 20E-promoted pupation, dsRNA targeting *DopEcR* was injected into the sixth instar 6 h larval hemocoel to knock down *DopEcR* and was followed by 20E induction. *DopEcR* was knocked down significantly in the larval epidermis, midgut, fat body, and head compared with the level of *dsGFP* (Fig 4A). After knockdown of *DopEcR*, the larvae showed delayed pupation, even after 20E injection, compared with the larvae that received *dsGFP* plus 20E (Fig 4B). Statistical analysis showed that 20E injection accelerated the initiation time of pupation by 29 h on average. However, pupation time was delayed by 43 h after injection of *dsDopEcR* plus 20E injection, compared with *dsGFP* plus 20E injection (Fig 4C). After knockdown of *DopEcR* and injection of 20E, the survival rate was 83%, the normal pupation rate was 9%, and the delayed pupation rate was 74%, with a significant difference compared with that for *dsGFP* plus 20E control (*p* < 0.01) (Fig 4D). These data suggested that DopEcR has a role in 20E-regulated pupation.

Terminal deoxynucleotidyl transferase nick-end-labeling (TUNEL) staining and histochemical analyses were performed to show the involvement of DopEcR in 20E-induced apoptosis of the larval midgut and fat body. Red fluorescence was observed in the larval midgut, which was separated from the imaginal midgut in the *dsGFP*+20E-treated larvae, whereas red fluorescence was not detected, and the imaginal midgut was not formed in the *dsDopEcR*+20E-treated larvae. Similarly, the fat body of the *dsGFP*+20E control larvae showed apoptosis signals and initial degradation, while the fat body of the *dsDopEcR*+20E-treated larvae was still closely arranged and did not exhibit obvious apoptosis signals (Fig 4E and 4F). These data suggested that DopEcR plays a role in 20E-induced apoptosis.

### 20E antagonizes dopamine function

**Fig 5:**
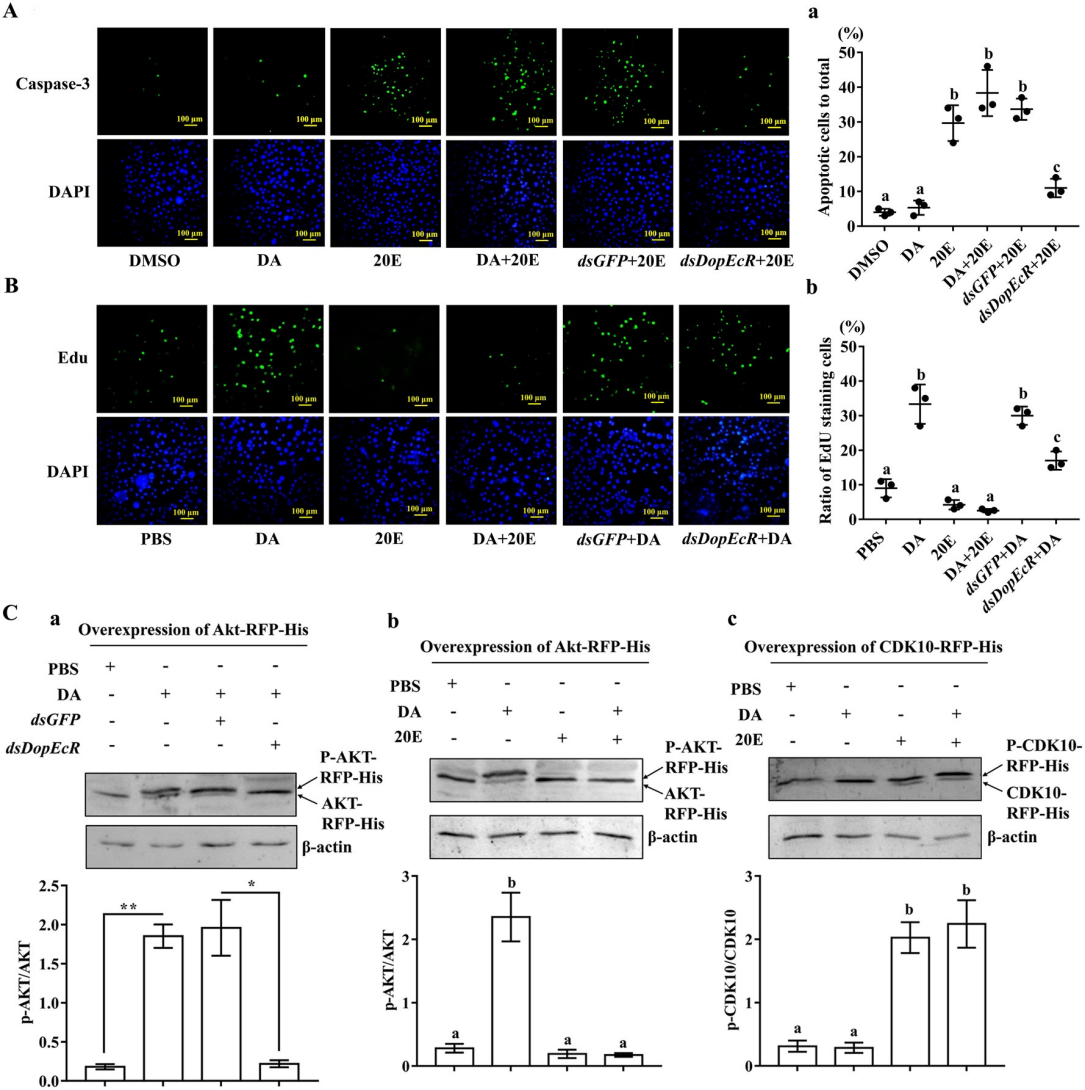
20E antagonizes dopamine function. **A**. Apoptosis signal in HaEpi cells after DMSO, 20E (5 μM), *dsGFP*+20E (5 μM), *dsDopEcR+*20E (5 μM) and DA (10 μM) +20E (5 μM) treatment by using the NucView caspase-3 activity assay kit. **a**. The ratio of apoptotic cells (green) to the total cells (blue) in the field view was obtained. **B**. Proliferation signal in HaEpi cells after DMSO, 20E (5 μM), *dsGFP*+20E (5 μM), *dsDopEcR+*20E (5 μM) and DA (10 μM) +20E (5 μM) treatment by using the 5-ethynyl-2′-deoxyuridine (EdU) kit (Ribobio, Guangzhou, China). **b**. The ratio of proliferation cells (green) to the total cells (blue) in the field view was obtained. DAPI stained the nucleus (blue). Statistical analysis using the data from 100 × 3 cells. The yellow bars represent 100 μM. **C**. Western blot analysis of 20E (5 μM) or DA (10 μM) induced proteins phosphorylation for 30 min. **a. b**. and **c**. AKT-RFP-His, AKT -RFP-His, and CDK10-RFP-His. 7.5% SDS-PAGE gel. Error bars show the mean ± SD of three times’ biological repetition. ImageJ software was used to transform the image data. Significant differences were calculated by Student’s *t* test (*p < 0.05; **p < 0.01) or one-way analysis of variance (ANOVA, *p* < 0.05).

The finding that 20E repressed larval feeding activity and thus induced earlier pupation prompted us to investigate the relationship between 20E and dopamine (DA), as well as the roles of DopEcR in the 20E and DA pathways by studying apoptosis and cell proliferation. Compared with cells treated with DMSO and DA, caspase-3 activity was detected in 31% of HaEpi cells (*H*. *armigera* epidermal cells) after the addition of 5 μM 20E for 72 h, suggesting that 20E induces apoptosis. However, caspase-3 activity was significantly reduced in HaEpi cells treated with *dsDopEcR* and 5 μM 20E, compared with that in *dsGFP* and 20E treated cells (Fig 5A and 5a), suggesting that 20E induces apoptosis via DopEcR. In contrast, the proliferative signal 5-ethynyl-2'-deoxyuridine (EdU) was detected in 33% of DA (10 μM)-treated cells, compared with that in the PBS-treated cells, suggesting that DA (10 μM) promotes cell proliferation. However, a low EdU signal was detected in the *dsDopEcR*+DA (10 μM)-treated cells compared with those treated with *dsGFP*+DA (10 μM). 20E did not induce a proliferative signal but repressed the DA-induced cell proliferation (Fig 5B and 5b) suggesting that DA promotes cell proliferation via DopEcR, and 20E opposes DA’s function.

Phosphorylation of protein kinase B (AKT) and CDK10 were examined to further confirm the antagonism of 20E and DA functions. Western blotting showed DA (10 μM) induced the phosphorylation of AKT, but knockdown of *DopEcR* blocked DA-induced AKT phosphorylation significantly (Fig 5Ca), suggesting that DA induces AKT phosphorylation via DopEcR. 20E (5 μM) repressed DA-induced AKT phosphorylation; however, DA did not repress 20E-induced CDK10 phosphorylation (Fig 5Cb and 5Cc). These results further confirmed that 20E antagonizes DA’s function.

### DopEcR is involved in the 20E pathway

**Fig 6:**
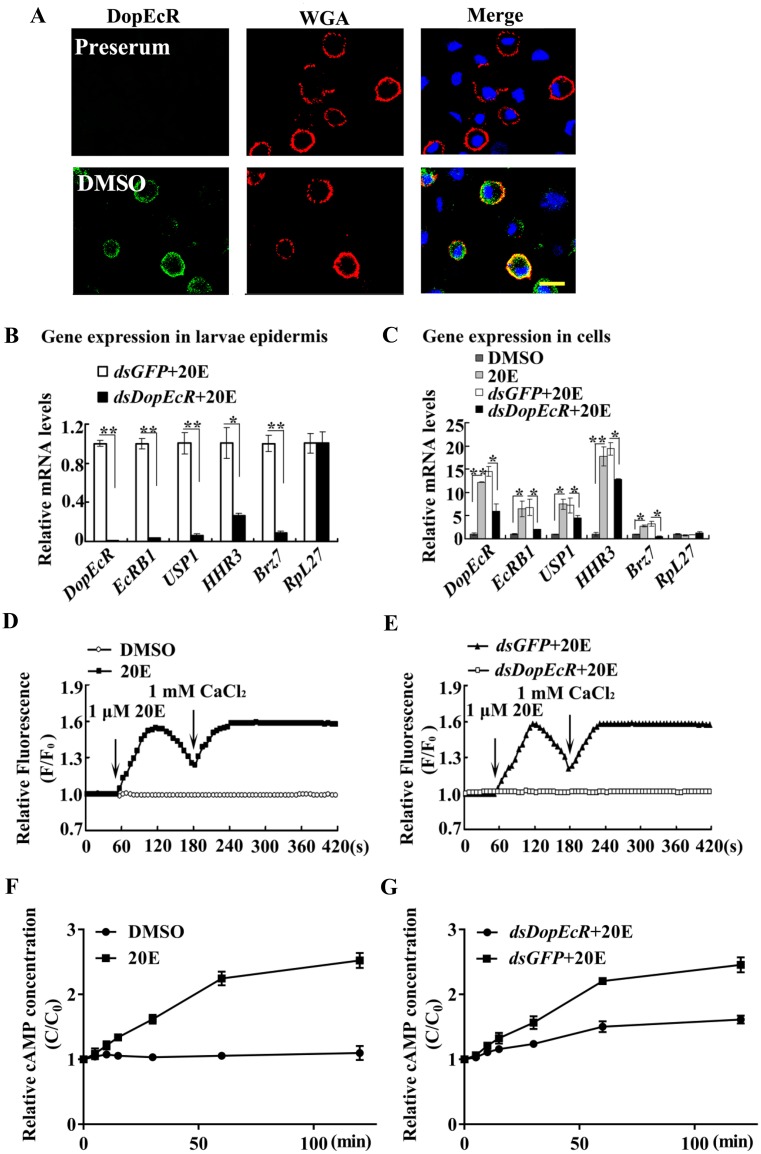
Roles of DopEcR in 20E pathway. **A**. DopEcR was localized in plasma membrane. **Red**: The cell membrane was marked by wheat germ agglutinin (WGA). **Green**: DopEcR protein stained with an anti-DopEcR antibody and secondary antibody labeled with Alexa-488. **Blue**: nucleus stained with 4’-6-diamidino-2-phenylindole dihydrochloride (DAPI). Observed by confocal microscope. Scale bar = 25 μm. **B**. and **C**. qRT-PCR showing mRNA levels of 20E-response genes after *DopEcR* knockdown in 6th-72 h larval epidermis (500 ng dsRNA/larva) and in HaEpi cells (2 μg dsRNA/mL, 48 h once, followed 1 μM 20E for 12 h). *β-actin* was regard as control. **D** and **E**. Ca^2+^ levels by Image Pro-Plus analysis after *DopEcR* knockdown in HaEpi cells, which representing three repeats. Cells were treated with *dsGFP* (2 μM) and *dsDopEcR* (2 μM) for 48 h and AM ester calcium crimson dye (3 μM) in DPBS for 30 min, and then by 20E (1 μM) and CaCl_2_ (1 mM), respectively. F: fluorescence intensity of HaEpi cells after different treatments. F_0_: fluorescence intensity before different treatments. Fluorescence was recorded per 6 s by confocal microscope photographs at 555 nm wavelength laser. **F**. and **G**. *DopEcR* knockdown repressed the 20E-triggered cAMP increase. HaEpi cells were transfected with *dsDopEcR* or *dsGFP* for 48 h followed by incubation with 2 μM 20E. Error bars showed the mean ± SD of three times repetition. Asterisks manifest significant differences by Student’s *t* test (*p < 0.05; **p < 0.01).

The subcellular location of DopEcR was analyzed to confirm that it is a cell membrane protein. DopEcR was localized in the plasma membrane in the DMSO solvent control by detection using antibodies against *H*. *armigera* DopEcR (Fig 6A); however, DopEcR was not internalized into the cytoplasm within 5 min to 1 h after 20E treatment (S1 Fig). These results suggested that DopEcR exerts its roles on the cell membrane.

To address the mechanism by which DopEcR functions in 20E-promoted pupation, the transcript levels of various 20E-responsive genes were examined after knockdown of *DopEcR* by injecting of *dsDopEcR* in to 6th instar 6 h larval hemocoel. The transcript levels of key genes in the 20E pathway, including ecdysone nuclear receptor *EcRB1*, heterodimeric partner *USP1*, and transcription factors *HHR3* and *BrZ7*, were decreased significantly in larval epidermis after knockdown of *DopEcR*, compared with that in the *dsGFP* control, with the housekeeping gene ribosomal protein *RpL27* as the internal reference (Fig 6B). Similarly, knockdown of *DopEcR* by incubating HaEpi cells with *dsDopEcR* decreased 20E-induced gene expression (Fig 6C). These data suggested that 20E induces gene expression via DopEcR.

20E regulates gene expression via a GPCR-mediated increase in the intracellular Ca^2+^ concentration to form the EcRB1/USP1 transcription complex [10]; therefore, we detected the involvement of DopEcR in 20E-induced Ca^2+^ levels. 20E induced rapid Ca^2+^ intracellular release and extracellular Ca^2+^ influx. However, after *DopEcR* knockdown, 20E could not induce rapid Ca^2+^ release and influx (Fig 6D and 6E). These data suggested that DopEcR participates in 20E-induced cellular Ca^2+^ increase.

A previous study showed that 20E triggers intracellular cAMP increase via ErGPCR-2 to enhance EcRB1/USP1-regulated gene transcription [12]. Therefore, the role of DopEcR in the 20E-induced increase in intracellular cAMP levels was detected in HaEpi cells. Compared with the DMSO-treated cells, the concentration of intracellular cAMP was increased by incubation with 20E (Fig 6F). However, cAMP concentrations decreased after *DopEcR* knockdown (Fig 6G). These results suggested that 20E increases the intracellular concentration of cAMP via DopEcR.

### DopEcR interacts with Gαq and Gαs and regulate protein phosphorylation

**Fig 7:**
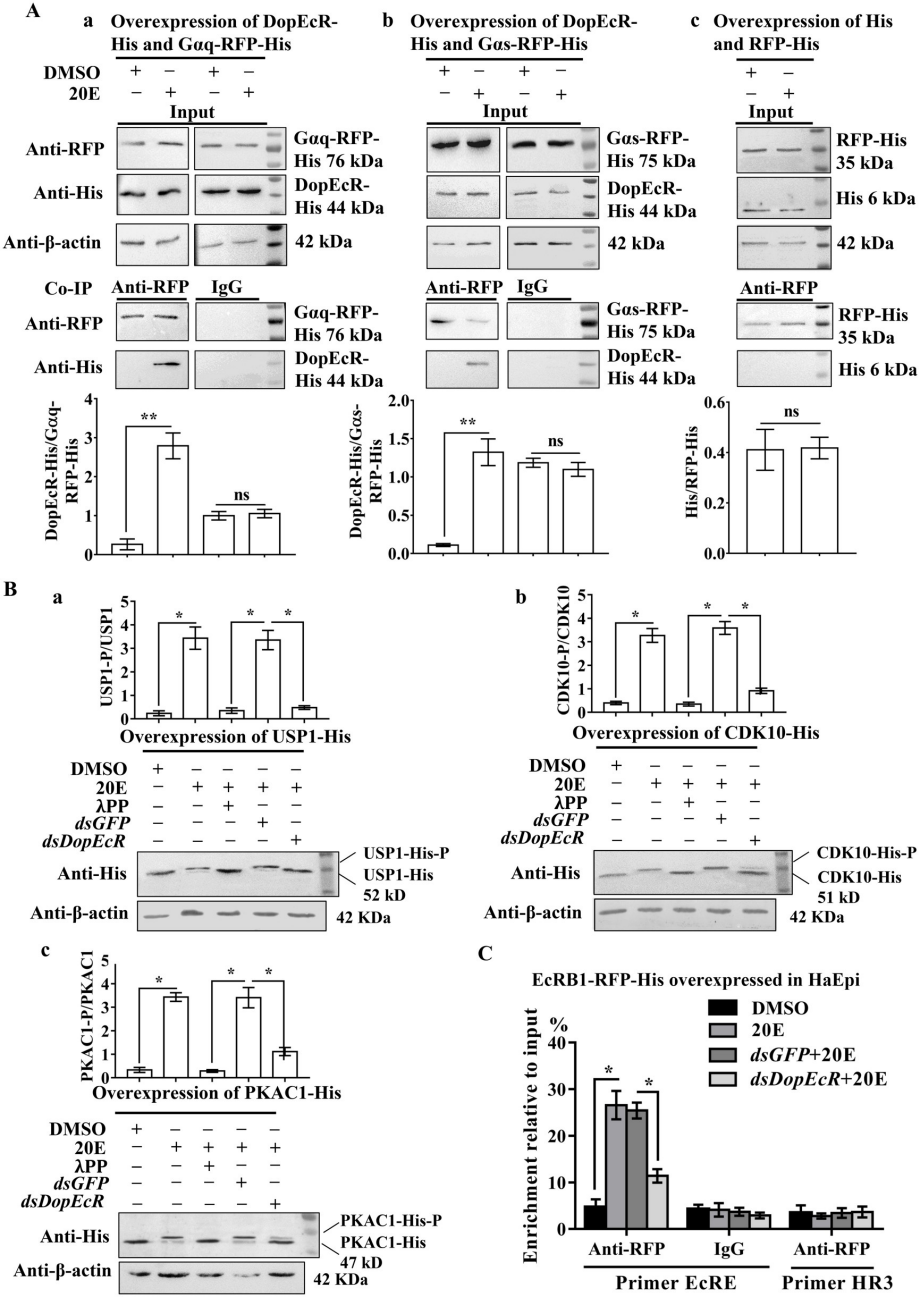
20E promotes DopEcR coupling with Gαq and Gαs and protein phosphorylation via DopEcR. **A**. Co-immunoprecipitation (Co-IP) to detect DopEcR coupling with Gαq and Gαs under 20E (2 μM for 30 min) induction. DMSO was solvent control. **a**. **b**. and **c. Input**: the levels of Gαq-RFP-His, DopEcR-His, Gαs-RFP-His and RFP-His in the cells detected by antibodies against RFP or His. β-actin was a loading control. **Co-IP**: Anti-RFP antibody co-immunoprecipitated Gαq-RFP-His and DopEcR-His, Gαs-RFP-His and DopEcR-His, or RFP-His and His. Nonspecific mouse IgG was a negative control. SDS-PAGE gel was 12.5%. **B**. Western blot analysis 20E-induced phosphorylation of proteins (2 μM 20E for 1 h). **a. b**. and **c**. USP1-His, CDK10-His and PKAC1-His. **λPP**: 0.5 μM λPPase incubation for 30 min at 30 °C. 7.5% SDS-PAGE gel. **C**. ChIP analyzing the involvement of DopEcR in 20E-induced EcRB1 binding to the EcRE. EcRB1-RFP-His was overexpressed in HaEpi cells for 48 h. The cells were incubated with *dsDopEcR* (2 μg/mL) or *dsGFP* (2 μg/mL) for 12 h, followed by inducing of 2 μM 20E or DMSO for 6 h. **Input**: non-immunoprecipitated chromatin. **IgG**: nonspecific mouse IgG. **Primer EcRE**: primers targeted to EcRE-containing DNA. **Primer HR3**: primers targeted to HR3 ORF. *p value via Student’s *t*-test based on three replicates in all figures (*p < 0.05; **p < 0.01).

Guanine nucleotide-binding protein subunit alpha S (Gαs) stimulates cAMP production and G protein subunit alpha Q (Gαq) promotes intracellular Ca^2+^ increase [30]. To address the mechanism by which 20E increases intracellular Ca^2+^ and cAMP levels via DopEcR, the protein interaction between DopEcR and Gαq or Gαs was examined by co-overexpression of DopEcR-His and Gαq-RFP-His or Gαs-RFP-His in HaEpi cells. RFP-His and His were overexpressed to exclude the possibility of protein interaction caused by the His- or RFP-His-tag. When Gαq-RFP-His and DopEcR-His were co-overexpressed in the input, DopEcR-His was precipitated together with Gαq-RFP-His under 20E induction using anti-RFP antibodies, but not in the negative control using IgG (Fig 7Aa). Similarly, DopEcR-His was precipitated together with Gαs-RFP-His using anti-RFP antibodies under 20E induction (Fig 7Ab). In the tag control, the His-tag was not precipitated together with RFP-His using the anti-RFP antibodies under 20E induction (Fig 7Ac). These data suggested that DopEcR directly interacts with Gαq and Gαs under 20E induction.

20E induces phosphorylation of USP1 and CDK10 via the ErGPCR-1-mediated PKC pathway for EcRB1/USP1 transcription complex formation and gene transcription [10, 11], and the ErGPCR-2-mediated PKA pathway induces phosphorylation of PKAC1 to enhance gene transcription in the 20E pathway [12]. Therefore, we examined the phosphorylation of these proteins to further reveal the involvement and mechanism of DopEcR in the 20E pathway. Western blotting showed that 20E induced USP1 phosphorylation. Lambda protein phosphatase (λPPase) treatment degraded the phosphorylation of USP1. However, *DopEcR* knockdown significantly suppressed 20E-induced phosphorylation of USP1, compared with that in the *dsGFP* control (Fig 7Ba). Similarly, *DopEcR* knockdown significantly suppressed 20E-induced CDK10 phosphorylation (Fig 7Bb) and PKAC1 phosphorylation (Fig 7Bc). These data suggested that 20E induces phosphorylation of these key proteins via DopEcR.

20E regulates gene transcription via the EcRB1/USP1 transcription complex binding to EcREs [4]. To address the role of DopEcR in 20E-induced gene transcription, we examined the binding of EcRB1-RFP-His to EcRE (GGGGTCAATGAACTG in the 5' regulatory region of *Helicoverpa HR3*) using a chromatin immunoprecipitation (ChIP) assay. The qRT-PCR results showed that EcRB1-RFP-His bound more EcRE under 20E treatment than in the DMSO treatment control, using the primers for EcRE located on the EcRE-containing DNA. However, EcRB1-RFP-His bound less EcRE in *dsDopEcR* treated HaEpi cells, compared with that in the *dsGFP*-treated cells. The IgG negative control and *HR3* primers located in the open reading frame (ORF) of *HR3* did not produce the same results (Fig 7C). These results suggested that 20E regulates EcRB1/USP1 transcription complex binding to EcRE via DopEcR.

### Modeling of DopEcR binding 20E

**Fig 8:**
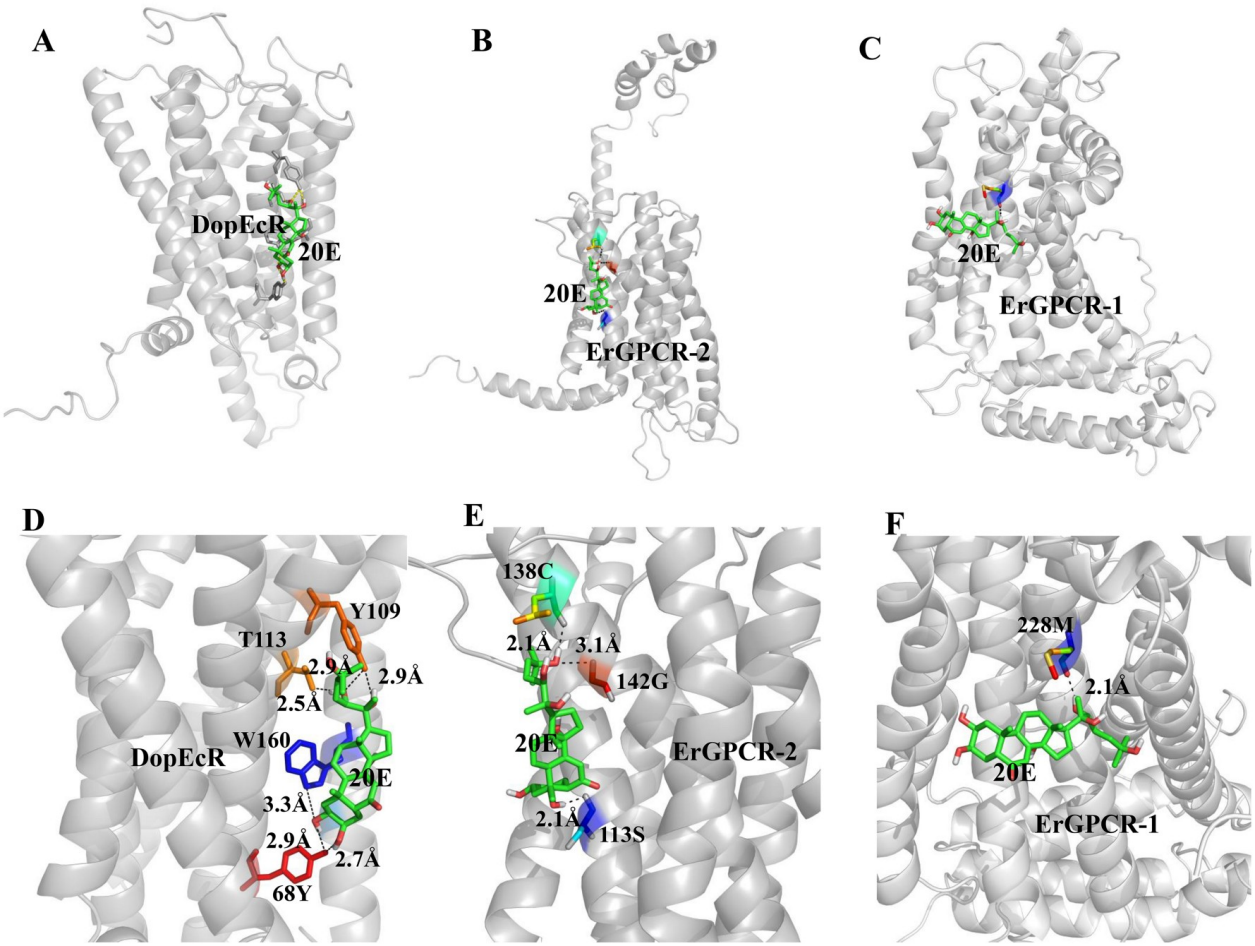
Modeling of the ligand-binding complex of the GPCRs. **A. B**. and **C**. Prediction of the Surflex-Dock (SFXC) program from the SYBYL X2.0 software. Overall structures of DopEcR, ErGPCR-2, and ErGPCR-1 (gray) and docked 20E (Green), respectively. **D. E**. and **F**. A closer view of the pockets relative to docking models of DopEcR-, ErGPCR-2- and ErGPCR-1-20E complex and the amino acid residues mutated in this study. The dotted lines indicate the predicted hydrogen bonds between the amino acid residues of GPCRs and 20E. Orientation is the same in all models.

Computational docking of 20E to DopEcR was conducted using Surflex-Dock (SFXC) in the SYBYL X2.0 software (Certara, Princeton, NJ, USA) to predict the possibility of DopEcR binding to 20E. ErGPCR-1 [8] and ErGPCR-2 [9] were analyzed to compare the results. The models led to highest score of 20E binding DopEcR, ErGPCR-2, and ErGPCR-1 near the transmembrane helix (Fig 8A–8C). 20E formed six hydrogen bounds with Tyr-68, Tyr-109, Thr-113, and Trp-160 of DopEcR; three hydrogen bonds with Cys-13, Ser-113, and Gly-142 of ErGPCR-2; and one hydrogen bond with Met-228 of ErGPCR-1 (Fig 8D–8F). The scores for DopEcR, ErGPCR-2, and ErGPCR-1 binding to 20E were 2.6433, 1.6124, and −0.5007, respectively, in which the higher the score, the stronger the combining ability. These results predicted that DopEcR and ErGPCR-2 have a higher binding ability to 20E than ErGPCR-1.

### DopEcR binds 20E

**Fig 9:**
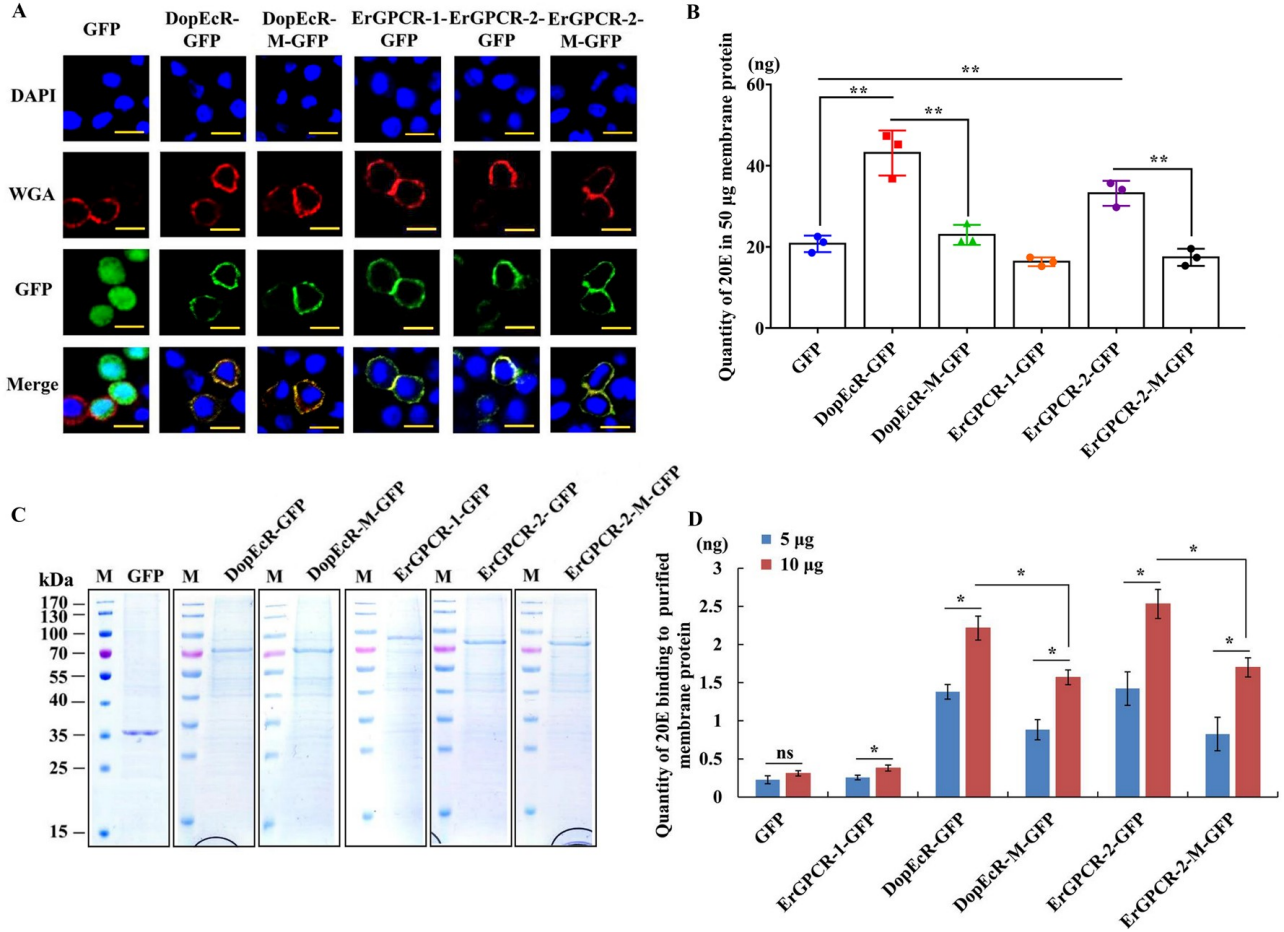
Detection of 20E that was bound by the GPCRs-overexpressing cell membrane proteins and the isolated GPCRs. **A**. Cell membrane localization of overexpressed GFP and GPCRs. Red: the cell membrane was marked by wheat germ agglutinin (WGA). Green: green fluorescence from GFP and various GPCRs fused with GFP. Blue: nucleus stained with 4’-6-diamidino-2-phenylindole dihydrochloride (DAPI). Observed by confocal microscope. Scale bar = 20 μm. **B**. Quantity of 20E bound by 50 μg membrane proteins from HaEpi cells that was overexpressing GFP, DopEcR-GFP, DopEcR-M-GFP, ErGPCR-1-GFP, ErGPCR-2-GFP, and ErGPCR-2-M-GFP. **C**. SDS-PAGE shows the partially purified GPCRs with Coomassie brilliant blue staining used in the experiments in D. **D**. Quantity of 20E bound by DopEcR-GFP, DopEcR-M-GFP, ErGPCR-2-GFP, and ErGPCR-2-M-GFP. Error bars represent the SD of three replicates. Asterisks indicate significant differences according to Student’s *t-*tests (*p < 0.05; **p < 0.01).

To prove that DopEcR binds 20E, DopEcR-GFP and its mutant DopEcR-M-GFP, which was mutated for possible steroid binding sites (S1 Table) based on the prediction of the Surflex-Dock (SFXC) program from the SYBYL X2.0 software (Certara, Princeton, NJ, USA) and I-TASSER online at http://zhanglab.ccmb.med.umich.edu/I-TASSER/ (S2 Fig), were overexpressed in Sf9 cells. GFP was overexpressed as a tag control. ErGPCR-1 [8] and ErGPCR-2 [9] were overexpressed for comparison of the ability of different GPCRs to bind 20E. The overexpressed ErGPCR-1-GFP, ErGPCR-2-GFP, DopEcR-GFP, and their mutants, ErGPCR-2-M-GFP and DopEcR-M-GFP, were confirmed to be located in the cell membrane (Fig 9A). Binding assay by 20-hydroxyecdysone enzyme immunoassay (20E-EIA) showed that the amount of 20E bound by cell membrane from ErGPCR-2-GFP and DopEcR-GFP overexpressing cells increased significantly compared with that bound by the GFP overexpressing cells. However, the amount of 20E bound by cell membranes from ErGPCR-1-GFP, ErGPCR-2-M-GFP, or DopEcR-M-GFP overexpressing cells did not increase compared with that from GFP overexpressing cells (Fig 9B). These results suggested that DopEcR and ErGPCR-2 could bind 20E in the cell membrane.

ErGPCR-1-GFP, ErGPCR-2-GFP, DopEcR-GFP, and their mutants, ErGPCR-2-M-GFP and DopEcR-M-GFP, were partially purified to determine their binding capacity to 20E. SDS-PAGE with Coomassie brilliant blue staining showed the GPCRs were partially purified (Fig 9C). A binding assay using 20E-EIA showed that the GFP tag and the isolated ErGPCR-1-GFP bound 20E at a very low level. However, DopEcR-GFP bound 20E in a dose dependent manner, with 5 μg DopEcR-GFP in 50 μL EIA buffer binding 1.3 ng of 20E, and 10 μg of DopEcR-GFP in 50 μL EIA buffer binding 2.3 ng of 20E. Compared with wild-type DopEcR-GFP, the DopEcR-M-GFP mutant bound less 20E, with 0.8–1.6 ng of 20E per being bound by 5–10 μg of DopEcR-M-GFP in 50 μL of EIA buffer. Similarly, ErGPCR-2-GFP bound 1.4–2.5 ng of 20E per 5–10 μg of ErGPCR-2-GFP in 50 μL of EIA buffer. However, the ErGPCR-2-M-GFP mutant bound less 20E, with 0.7–1.7 ng of 20E being bound per 5–10 μg protein in 50 μL of EIA buffer (Fig 9D). These data suggested that both DopEcR and ErGPCR-2 could bind 20E after they were partially purified from the plasma membrane.

**Fig 10:**
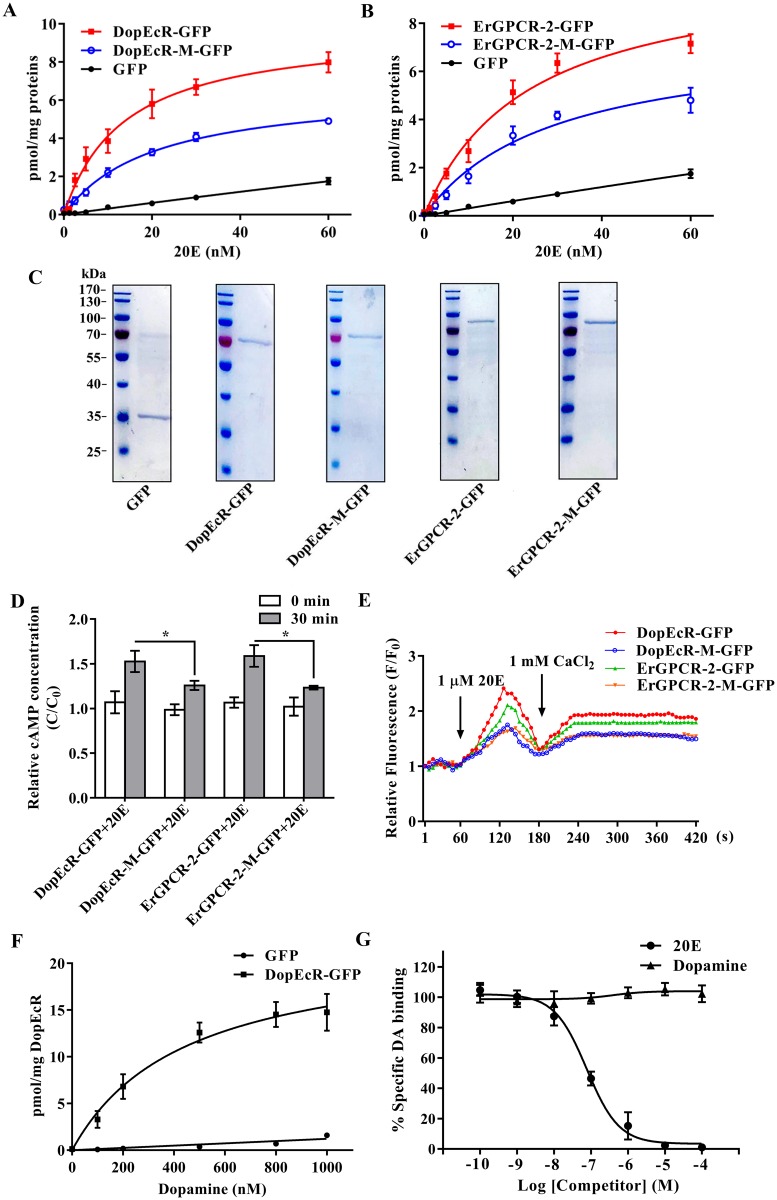
Affinity of GPCRs to 20E. **A**. Saturation binding curves of DopEcR-GFP and DopEcR-M-GFP to 20E. **B**. Saturation binding curves of ErGPCR-2-GFP and ErGPCR-2-M-GFP to 20E. Nonspecific binding was analyzed with GFP-His tag. All the experiments were performed using 10 μg of isolated protein in 50 μL EIA buffer. **C**. SDS-PAGE to show the highly purified GPCRs used in A, B, F and G. **D**. The cAMP levels of HaEpi cells overexpressed with DopEcR-GFP, DopEcR-M-GFP, ErGPCR-2-GFP, and ErGPCR-2-M-GFP under 20E-triggered. HaEpi cells were transfected with DopEcR-GFP, DopEcR-M-GFP, ErGPCR-2-GFP, and ErGPCR-2-M-GFP for 48 h followed by incubation with 2 μM 20E. **E**. Ca^2+^ levels after DopEcR-GFP, DopEcR-M-GFP, ErGPCR-2-GFP, and ErGPCR-2-M-GFP overexpressed in HaEpi cells. Cells were transfected with DopEcR-GFP, DopEcR-M-GFP, ErGPCR-2-GFP, and ErGPCR-2-M-GFP for 48 h and AM ester calcium crimson dye (3 μM) in DPBS for 30 min, and then by 20E (1 μM) and CaCl_2_ (1 mM), respectively. F: fluorescence intensity of HaEpi cells after different treatments. F_0_: fluorescence intensity before different treatments. Fluorescence was recorded per 6 s by confocal microscope photographs at 555 nm wavelength laser and then analyzed using Image Pro-Plus software. **F**. Saturation binding curves of DopEcR-GFP to DA. Nonspecific binding was analyzed with GFP-His tag. **G**. The competition curves of DopEcR-GFP to 20E and Dopamine. The ELISA plate coated with DopEcR-GFP was incubated with 20 pmol of DA in the presence of increasing concentrations of the different ligands (20E and DA). Error bars represent the SD of three replicates. Asterisks indicate significant differences according to Student’s *t-*tests (*p < 0.05; **p < 0.01).

A saturation binding curve was constructed using 20E-EIA to further examine the affinity of GPCRs to 20E by calculating the dissociation constants (Kd). The saturable specific binding of DopEcR-GFP to 20E had a Bmax of 9.764 ± 0.4953 pmol/mg protein and a Kd of 17.98 ± 3.005 nM. However, the DopEcR-GFP mutant decreased the saturable specific binding, with a Bmax of 6.661 ± 0.2764 pmol/mg protein and a Kd of 20.5 ± 1.98 nM (Fig 10A). In comparison, the saturable specific binding of ErGPCR-2-GFP to 20E had a Bmax of 10.42 ± 0.6629 pmol/mg protein and a Kd of 23.32 ± 3.304 nM. ErGPCR-2-GFP mutant decreased the Bmax to 7.5 ± 0.6592 pmol/mg protein and produced a Kd of 29.03 ± 5.275 nM (Fig 10B). The GPCRs used for saturation binding curve analysis were proven to be highly purified (Fig 10C). The 20E-EIA assay is based on competition between the unlabeled 20E (20E bound to GPCR) and AChE-labelled 20E (Tracer) for the limited-specific rabbit anti-20E antiserum; therefore, an inhibition or competitive curve was not detected. These data confirmed that the DopEcR-GFP and ErGPCR-2-GFP could bind 20E.

We further addressed the roles of DopEcR-GFP and ErGPCR-2-GFP in 20E pathway by examining cAMP and Ca^2+^ levels with 20E induction. The HaEpi cells that were overexpressing mutants DopEcR-M-GFP and ErGPCR-2-M-GFP had lower intracellular cAMP levels than that in DopEcR-GFP and ErGPCR-2-GFP overexpressing cells by 20E incubation (Fig 10D). Similarly, 20E induced more Ca^2+^ intracellular release and extracellular Ca^2+^ influx in HaEpi cells overexpressing DopEcR-GFP and ErGPCR-2-GFP compared with that in the cells overexpressing DopEcR-M-GFP and ErGPCR-2-M-GFP (Fig 10E). These data suggested that DopEcR and ErGPCR-2-GFP participate in the rapid cellular responses induced by 20E.

The competitive binding of DA and 20E to DopEcR was analyzed further to address the relationship of the two ligands to the same receptor. Based the typical kinetic and saturation binding curves simulated in GraphPad Prism using the association kinetics equation, DopEcR to DA had a Kd of 447.5 ± 100.6 nM (Fig 10F), which suggested that DopEcR could bind DA. The competition displacement binding curves were constructed at a ligand DA concentration equivalent to the Kd (450 nM) and various concentrations of ligand 20E. 20E displaced DA and the calculated inhibition constant (Ki) was 42.5 ± 8.6 nM for 20E (Fig 10G). 20E displacement studies indicated that 20E could displace DA from DopEcR. These data showed that receptor had a much higher affinity for 20E than for DA.

## Discussion

DopEcR can bind either DA or 20E; however, the outcomes and mechanisms are unclear. Whether GPCRs are steroid hormone receptors has not been determined because of a lack of direct evidence of GPCRs binding steroid hormones. The present study provided evidence that 20E binds to DopEcR to stop larval feeding and transmit the signal for insect pupation. Isolated GPCRs can bind 20E. 20E upregulates DopEcR expression to transmit the 20E signal for gene expression and lepidopteran insect pupation.

### 20E binds to DopEcR to stop larval feeding and transmit the signal for pupation

DA receptors play important roles in animal motor function and reward-motivated behaviors, including eating [21] and reward-induced eating behavior [22] in humans. The loss of dopamine neurons results in Parkinson’s disease [23] and Alzheimer’s Disease [31]. We observed that knockdown of *DopEcR* at the larval growth stage repressed larval feeding, suggesting that DopEcR plays role in larval feeding, and the function of the DA receptor in food consumption is conserved from insect to mammals. Dopamine has been linked to motivated behavior and rewarding reinforcement in fruit flies [32]. Dopamine-receptor signaling pathways play roles in olfactory associations in *Drosophila* [33]. The repression of DA function might effect the decrease of motor function or reward-motivated behaviors and therefore decrease feeding.

20E reduces food consumption in *Bombyx mori* [34], and initiates wandering behavior in *Manduca* by directly acting on the central nervous system in the brain [35]; however, the mechanism is unclear. We observed that 20E repressed larval feeding and promoted earlier pupation. 20E can bind DopEcR to repress the DA pathway. The 20E titer in *H*. *armigera* increased from 0.5 μM to 4.7 μM from 6th instar 48 h feeding larvae to 6th instar 120 h wandering larvae. Ecdysteroids have a much higher affinity to DopEcR compared with DA, and the larger size of ecdysteroids excludes DA binding to the receptor and inhibits the DA-mediated increase in cAMP levels [16]. The relationship between 20E and DA through the DopEcR is only verified in CHO cells and Sf9 cells [16]. In addition, DopEcR mediates the non-canonical actions of 20E and rapidly modulates *Drosophila* adult conditioned behavior such as courtship memory through cAMP signaling [28]. In this study, we proposed that 20E blocks larval feeding by competitively binding to DopEcR for 20E signaling. Therefore, larvae stopped feeding and initiated pupation when DopEcR was increased at the metamorphic stages in 6th instar larvae. The decrease of the DA titer before pupation was possible due to the degeneration of the larval brain. The increase of the DA titer during pupal stage was possible related to the remodeling of adult brain for pupal motion, which needs further study.

*Drosophila* DopEcR mutants (hypomorphic alleles) can reach adulthood normally. DopEcR functions as a nongenomic ecdysone receptor to control experience-dependent courtship suppression in mushroom body neurons [28], showing DopEcR mutation is not lethal. In our study, the knockdown of *DopEcR* caused delayed pupation, repressed larval feeding, growth, body weight, and low death rate, suggesting that DopEcR plays role in larval feeding during growth stage, and functions as 20E receptor during metamorphosis.

GPCRs interact with Gαs and Gαq to increase intracellular cAMP and Ca^2+^ to activate the PKA and PKC pathways, respectively [36]. The GPCR-cAMP-PKA and GPCR-Ca^2+^-PKC pathways are involved in animal steroid hormone non-genomic pathway signaling [37]. Acute 20E feeding induces a rapid increase in cAMP levels in the MBs via DopEcR, and DopEcR modulates the adult conditioned behavior through cAMP signaling, thus mediating the non-canonical actions of 20E, such as behavioral control [28]. 20E-induced cAMP increases and PKAC1 phosphorylation regulates the phosphorylation of cAMP-response element binding protein (CREB), which binds to the cAMP response element (CRE) to enhance 20E-regulated gene expression for pupation in *H*. *armigera* [12]. We observed that DopEcR interacts with Gαs and Gαq directly under 20E induction. 20E increased cAMP and Ca^2+^ levels via DopEcR, induced protein phosphorylation of USP1, CDK10, and PKAC1, and increased the binding of EcRB1 to EcRE. GPCRs transmit the 20E signal in the cell membrane in *H*. *armigera* [8, 9]. Non-genomic GPCRs, Gαq, phospholipase C (PLC) 1, Ca^2+^, and the protein kinase C (PKC) signaling cascade have been identified in *H*. *armigera*, suggesting that activation of the PKC pathway is necessary for USP phosphorylation and that 20E-responsive gene transcription occurs in the 20E genomic pathway [10, 38]. 20E triggers lysine acetylation of USP1 by activating the GPCR, PLC1, Ca^2+^, and CaMKII signaling pathways, which is essential for the formation of the EcRB1-USP1 transcription complex and gene transcription [13]. ErGPCR-2 knockdown blocks the 20E-induced calcium increase and phosphorylation of USP1 and CDK10, which represses the formation of the 20E transcription complex EcRB1/USP1 [9]. 20E regulates heterodimeric partner (USP1) phosphorylation via phospholipase C-gamma-1 (PLCG1) and connects the GPCR-mediated non-genomic pathway to the nuclear receptor EcRB1-mediated genomic pathway [9, 10]. Our data confirm the role of DopEcR in the 20E signaling pathway; therefore, knockdown of *DopEcR* at later larval stages represses pupation. This is the first identification of the dual functions and mechanism of DopEcR, being either involved in larval feeding or in the 20E pathway for insect pupation.

### GPCRs can bind 20E

It has been suggested that GPCRs are able to bind steroid hormones in *Drosophila* [16] and in mammals [15] using cells or cell membranes that overexpress GPCRs. The binding affinities of steroid membrane receptors are orders of magnitude lower than those of nuclear receptors [39]. In addition, the binding affinity of the analog of 20E, [^3^H]Pon A, to the nuclear receptor complex of EcR/USP is one to two orders of magnitude higher than that of 20E [40]. The Kd value of the *in vitro* translated EcR/USP heterodimer for Pon A was 0.9 nM in *Drosophila* [41] and 1.1 nM in *Bombyx* [42], respectively. However, the membranes of the anterior silk gland of *B*. *mori*, which contain a putative membrane receptor of ecdysone, mEcR, exhibit saturable binding for [^3^H]Pon A, with a Kd of 17.3 nM and a Bmax of 0.82 pmol∙mg^-1^ protein [7]. The membranes isolated from Sf9 cells that overexpressed DmDopEcR showed saturable specific binding for [^3^H]Pon A, with a Kd of 10.4 ± 0.38 nM and a Bmax of 0.32 ± 0.04 pmol∙mg^-1^ protein [16].

In this study, we used the 20E-EIA method to detect isolated GPCRs binding 20E directly. We detected that isolated DopEcR exhibited saturable specific binding for 20E, with a Kd of 17.98 ± 3.005 nM and a Bmax of 9.764 ± 0.4953 pmol/mg protein. ErGPCR-2 showed saturable specific binding for 20E, with a Kd of 23.32 ± 3.304 nM and a Bmax of 10.42 ± 0.6629 pmol/mg protein. The Kd values of DopEcR and ErGPCR-2 have the same orders of magnitude as the Kd detected from the membranes of Sf9 cells, however, the Bmax values of DopEcR and ErGPCR-2 were higher than those detected from the membranes of Sf9 cells. A higher Bmax suggests a higher binding capacity of GPCRs than the membranes of Sf9 cells. This may be due to the different detection methods used, in which we used the 20E-EIA method and isolated GPCRs. The Kd values of isolated DopEcR for 20E (17.98 nM) in *H*. *armigera* is higher than that of EcR/USP heterodimer for Pon A (0.9 nM) in *Drosophila* [41], suggesting lower binding affinity of GPCRs to 20E than nuclear receptor, which might be one of the reasons that multiple GPCRs, such as DopEcR and ErGPCR-2, are involved in 20E signaling on the cell membrane. However, 20E shows higher affinity for DopEcR than DA and can inhibit the function of DA, and induces rapid signaling effects, such as increased Ca^2+^ and cAMP levels to trigger 20E-pathway.

Previous studies demonstrated that ErGPCR-1 and ErGPCR-2 transmit 20E signals in the lepidopteran insect *H*. *armigera*, but could not detect the binding of ErGPCR-1 and ErGPCR-2 to the 20E analog [^3^H]Pon A using the isotope method [8, 9]. In the present study, we confirmed that ErGPCR-2 can bind 20E using the 20E-EIA method. However, binding of ErGPCR-1 to 20E was not detected in this study. Agonists that exert their roles without measurable binding have been observed in another study [43]. A possible reason is that GPCRs loosely or dynamically binds to the ligands [44, 45].

### 20E upregulates DopEcR expression

In humans, dopamine receptors regulate various physiological processes, including reward, voluntary movement, and hypertension [46]. Insufficient or hyperactive dopaminergic signaling results in disease [23]. Thus, dopamine receptors are common neurological drug targets [47]. Dopaminergic neurotransmitters have been used to treat mental and neurological diseases, such as schizophrenia, bipolar disorder, Parkinson’s disease, Huntington’s disease, and Tourette’s syndrome [48]. Steroid hormone estrogens can regulate neurotransmission in the human central nervous system, including mood, reward, and motivation [49]. We observed that 20E upregulated DopEcR expression and bound to DopEcR to block its activity of DA pathway. In addition to insects, various plants produce 20E, such as *Cyanotis vaga*. Our finding presents the possibility to explore the use of 20E or other phytosterols to treat dopamine-related diseases.

### Various GPCRs transmit the 20E signal

*H*. *armigera* DopEcR is a class A (Rhodopsin-like) receptor that shares 68% identity with *Drosophila* DmDopEcR. ErGPCR-1 and ErGPCR-2 belong to class B (secretin receptors) (S3 Fig). However, *H*. *armigera* DopEcR showed increased expression levels during metamorphosis, which is different from DmDopEcR, which is rarely expressed in third-stage larvae, but is strongly expressed in the first and second larval stages and in adult heads [16], which suggested a difference between dipteran and lepidopteran insects. 20E induces a rapid increase in cAMP levels in MBs and triggers cAMP signaling via DopEcR to modulate adult conditioned behavior in *Drosophila* [28]. DopEcR is mainly expressed in the brain; however, ErGPCR-1 and ErGPCR-2 are expressed widely in the epidermis, fat body, and midgut [8, 9]. DopEcR is localized in the plasma membrane and is not internalized, which is similar to ErGPCR-1 [8], but differs from ErGPCR-2, which can be internalized after phosphorylation by GPCR kinase 2 (GRK2) to desensitize 20E signaling [9]. The different expression levels of GPCRs in tissues might explain why various GPCRs transmit 20E signals. That different GPCRs recognize unique positions of the G-protein barcode [50] might also explain why various GPCRs are involved in the same pathway.

### Conclusion

**Fig 11:**
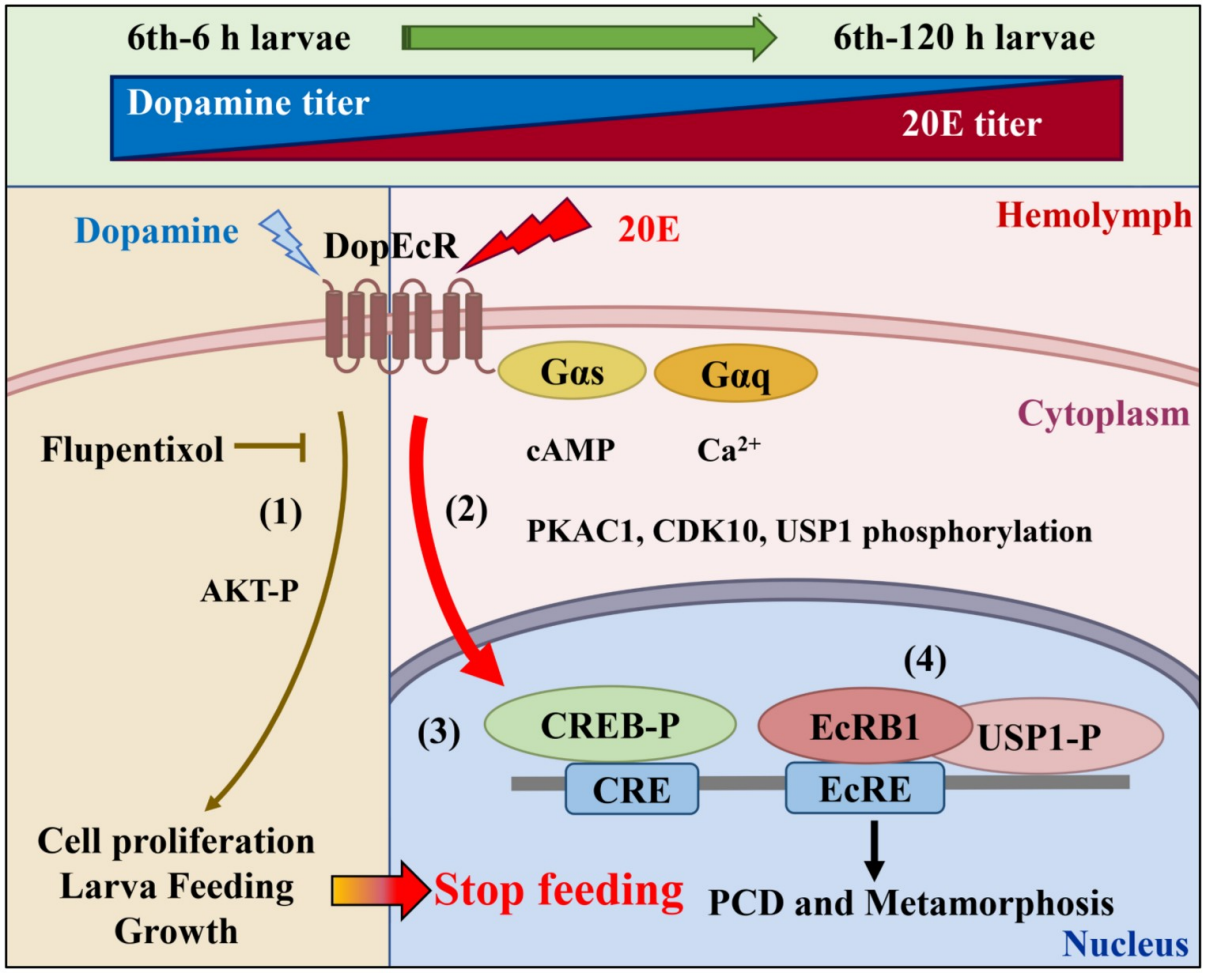
Diagram illustrating the roles of DopEcR in larval feeding and 20E-regulated metamorphosis. Dopamine titer in hemolymph decreased and 20E titer in whole larvae increased from 6 instar 6 h to 6 instar 120 h larvae. Dopamine binds DopEcR to promote AKT-P levels, larval feeding and cell proliferation (1). The increased 20E binds to DopEcR to initiate 20E signal, including the interaction of DopEcR and Gαs and Gαq, increase of cAMP and Ca^2+^ levels, and protein phosphorylation of PKAC1, CDK10 and USP1 (2). The phosphorylated-CREB (CREB-P) binds to CRE to promote 20E-regulated gene transcription [12] (3). The phosphorylated-USP1 and CDK10 forms EcRB1/USP1 transcription complex and bind to ecdysone response element (EcRE) to regulate gene expression for insect metamorphosis [10, 11] (4).

DopEcR functions as a DA receptor during the larval feeding stage to promote AKT phosphorylation, cell proliferation, larval feeding, and growth. 20E upregulates DopEcR expression during metamorphosis and binds to DopEcR to block its function in larval feeding and cell proliferation. 20E triggers the interaction of DopEcR with Gαq and Gαs, increases intracellular Ca^2+^ and cAMP levels, and induces protein phosphorylation to regulate 20E-pathway-induced gene expression to effect metamorphosis (Fig 11). GPCRs can bind 20E and function as 20E cell membrane receptors.

## Materials and methods

### Animals

The cotton bollworms, *Helicoverpa armigera*, which come from the Henan Agricultural University in China, were fed on artificial diet comprised of soybean powder, wheat germ with compound vitamins and mineral salt. And the insects were raised in an insectarium under the cycle of 14 h light: 10 h dark at 26±1 °C with 60% to 70% humidity.

### Preparation of rabbit polyclonal antibodies against DopEcR

A cDNA fragment (nucleotide sequence 148 bp to 1,020 bp, encoding 290 amino acids from 50 to 339) of *DopEcR* was connected with pET30a and expressed in *Escherichia coli* BL21 (DE3). The recombinant DopEcR protein formed occlusion bodies and was purified by means of 12.5% SDS-PAGE. The recombinant DopEcR protein was cut from the gel for preparing rabbit polyclonal antiserum [8].

### Western blotting

Dissected age-appropriate larvae, epidermis, midgut, fat body and brain and homogenized in 500 μL Tris-HCl buffer (40 mM, pH 7.5) on ice with 5 μL phenylmethylsulfonyl fluoride (PMSF, 17.4 mg/mL in isopropyl alcohol), respectively. Homogenate was centrifuged for 10–15 min at 4 °C at 12,000 rpm. And then the supernatant was collected. The protein concentration was measured by Bradford protein assay [51]. 20 μg proteins were separated by 7.5% or 12.5% SDS-PAGE and transferred to a nitrocellulose membrane. The membrane was blocked by blocking buffer [Tris-buffered saline (TBS, 150 mM NaCl solution, 10 mM Tris-HCl, pH 7.5) with 2–5% skim milk] for 1 h at room temperature. The membrane was incubated in blocking buffer with primary antibodies at 4 °C overnight. The polyclonal antibodies against *H*. *armigera* DopEcR were produced in our laboratory. Antibody against *H*. *armigera* β-actin, His, GFP, and RFP was purchased from ABclonal (Cat. AC026, AE003, AE012, and AE020, Wuhan, China). Antibodies against DopEcR, β-actin, His, GFP, and RFP were diluted in 5% skim milk at 1:5,000–1:10,000. After being washed three times with TBST (0.02% tween in TBS) for 10 min each, the membrane was incubated with 1:10,000 diluted secondary antibody, alkaline phosphatase-conjugated AffiniPure Goat Anti-Rabbit/-Mouse IgG (ZSGB-BIO, Beijing, China). After being washed three times with TBST and TBS, the target signals were visualized in 10 mL TBS, 45 μL of P-nitro-blue tetrazolium chloride (NBT, 75 μg/μL) and 30 μL of 5-bromo-4-chloro-3 indolyl phosphate (BCIP, 50 μg/μL) in the dark for 10–20 min. The membrane was washed by deionized water and made image by software of Photoshop. Bands on the membrane were calculated by software of ImageJ (National Institutes of Health, http://imagej.nih.gov/ij/download.html). Acquired data were analyzed by software of GraphPad Prism 7 (GraphPad Software, San Diego, CA, USA).

### Detection of the 20E and dopamine titer

More than three larvae, pupae, or adults at different developmental stages were collected and weighed. The whole bodies were then freeze-dried for 16 h. Then, 80% methanol was used to grind larvae, pupae, or adults from at least three insect samples at 4 °C to extract the 20E, 1 g tissues per 1 mL methanol. The sample was centrifuged at 10,000 × *g* for 10 min at 4 °C, and the supernatant was collected and evaporated until it dried completely. The pellet was dissolved in 1 mL EIA buffer and diluted 1,000 times. A 50 μL sample was used to detect 20E using a 20E Enzyme Immunoassay (20E-EIA) kit (Bertin Pharma, France) according to the instruction and operation manual.

The hemolymph samples collected from larvae, pupae, or adults were immediately transferred to a 1.5 mL polypropylene micro-test tube with 0.1 mM Phenylthiourea (PTU), respectively. The sample was centrifuged at 20,000 *g* for 10 min at 4 °C. The supernatant was detected the concentration of DA directly using an insect hemolymphal dopamine ELISA kit (MLBIO Biotechnology, Shanghai, China) according to the instruction and operation manual.

### Hormonal treatment of *H*. *armigera* larvae

20E was dissolved in dimethyl sulfoxide (DMSO) to prepare a 10 mg/mL stock solution. The work solution of 20E was diluted with sterile phosphate-buffered saline (PBS, pH 7.4, 10 mM sodium phosphate, 140 mM NaCl) at 1:100. The 6th instar 6 h larvae were selected and injected with 20E at 500 ng/5 μL per larva. The equivalent amount of DMSO was injected into the same stage larvae as control. The larvae were suffered at 1, 3, 6, 12, and 24 h, respectively after injected 20E or DMSO. The total mRNA or protein was extracted for qRT-PCR or western blot. The data of qRT-PCR were analyzed by software of GraphPad. The bands of western blot were calculated by software of ImageJ and the acquired data were analyzed by software of GraphPad.

### Quantitative real-time reverse transcription-polymerase chain reaction (qRT-PCR)

The total RNA was extracted with Trizol reagent according to the manufacturer’s instructions (TransGen Biotech, Beijing, China), and the first-strand cDNA was synthesized using M-MLV reverse transcriptase according to the manufacturer’s instructions (TIANGEN, Beijing, China). qRT-PCR was performed with a CFX96 real-time system (Bio-Rad, Hercules, CA, USA) using 2×SYBR qRT-PCR pre-mixture (TransGen Biotech, Beijing, China) and the used primers were listed in S2 Table. The relative expression levels of genes were obtained using *H*. *armigera β-actin* as a quantity control. The data from three independent experiments and three technique repeats were obtained by R = 2^−ΔΔCT^ method (ΔΔCT = ΔCT_sample_-ΔCT_control_, ΔCT = CT_gene_-CT_actin_) [52].

### Double-stranded RNA synthesis

RNA interference (RNAi) was used to study the DopEcR function because RNAi was widely used in many species of moths in 10 families [53]. Long dsRNA can be processed into smaller fragments [54] to suppress transcription of the target gene in worms [55]. The total RNA was extracted using Trizol reagent on the basis of the manufacturer’s instruction (TransGen Biotech, Beijing, China), and the first-strand cDNA (301 bp) from 297 bp to 598 bp was synthesized with HaDopEcR-RNAiF/ HaDopEcR-RNAiR (S2 Table) and M-MLV reverse transcriptase on the basis of the manufacturer’s instructions (TIANGEN, Beijing, China). Transcription was carried out as follows: 1 μg cDNA template was mixed with 20 μL 5 × transcription buffer, containing 80 U T7 RNA polymerase (Fermentas, Los Angeles, USA), 2.4 μL 10 mM A/U/C/GTP each (Fermentas, Los Angeles, USA), and 120 U RNasin (TaKaRa Bio, Otsu, Shiga Prefecture, Japan). RNase-free water was added to a volume of 50 μL. After incubation at 37 °C for overnight, 10 μL 40 U RNase-free DNase I (Fermentas, Los Angeles, USA), 10 μL DNAase I Buffer and 30 μL RNase-free water were added and the solution was incubated at 37 °C for 60 min. After extraction with phenol/chloroform and precipitation with ethanol, dsRNAs were resuspended with 50 μL RNase-free water. The purity and integrity of dsRNAs were determined using agarose gel electrophoresis. The dsRNAs were quantified by a Micro-Spectrophotometer (GeneQuant; Amersham Biosciences).

### RNA interference in larvae by feeding *Escherichia coli* (HT115/DE3)

RNAi was performed according to our previous article [56]. The continuous ingestion of the bacteria HT115/DE3 containing dsRNAs expressed shows higher interference efficiency compared with one-time injection of *H*. *armigera* larvae with dsRNA [57]. Interfering primers (HaDopEcR-swRNAiF/HaDopEcR-swRNAiR) (S2 Table) were used for PCR amplification of the target fragment (301 bp cDNA, from 297 bp to 598 bp). The target fragment was inserted into pPD129.36 (L4440) vector presented by Dr. Marek Jindra (Biology Center ASCR, Czech Republic). The vector was transformed into DH5α, and positive clones were screened using 50 μL ampicillin (Amp, 25 mg/mL). The recombinant plasmid was transformed into HT115 which provided by Dr. Marek Jindra (Biology Center ASCR, Czech Republic). Positive clones were screened with 50 μL Amp (25 mg/mL) and 6.25 μL tetracycline (Tet, 25 mg/mL) and inoculated into 5 mL medium (yeast extract 0.5 g, tryptone 1 g, NaCl 1 g, ddH_2_O 100 mL, pH 7.4) on programmable incubator shaker at 37 °C overnight. Overnight strains were inoculated into 100 mL medium at a volume of 1: 100 and shaken culture 3 h to OD_600_ = 0.4. Isopropyl β-D-1-thiogalactopyranoside (IPTG, 0.5 mM) was added in medium. Bacteria were cultured on programmable incubator shaker at 37 °C for 4 h. Bacterial RNA was extracted and dsRNA expression was detected by 2% agarose gel electrophoresis. The remaining bacteria were suspended in 400 μL PBS. Two groups of cotton bollworm were selected, 30 in each group. The bollworm food was cut into 1 cm **×** 1 cm **×** 0.2 cm. 10 μL fresh HT115 expressing *dsDopEcR* with PBS was applied to the feedstuff surface. Larvae were fed with HT115 every day up to 6th-96 h. Control groups were fed with feedstuff containing the same amount of *dsGFP* expressing HT115. Total RNA was extracted from the epidermis, midgut, head, and fat body of 6th 72 h instar, respectively. Interference efficiency was detected by qRT-PCR or western blot.

### RNA interference in larvae by injection of dsRNA

The dsRNAs were diluted with PBS to 100 ng/μL. When the 6th instar 6 h larva was stiff and numb on ice, 500 ng dsRNAs were injected into larval hemocoel from the third thoracic legs with a sterile microsyringe. dsRNAs were injected three times 24-hour intervals. In the third injection, 500 ng 20E and 500 ng dsRNAs were injected to larva together. The morphology and behavior of insects were observed since the first dsRNA injection.

### RNA interference in HaEpi cells

DNA fragments (300–500 bp) of GPCRs and green fluorescence protein (GFP) were PCR-amplified as templates for dsRNA synthesis with the primers (S2 Table). The cells of *H*. *armigera* epidermal cell line (HaEpi) were established from *H*. *armigera* epidermis and cultivated in Grace’s medium with 10% fetal bovine serum (FBS) at 27 °C [58]. When HaEpi cells density reached 70% to 80%, the cells were transfected with 2–4 μg dsRNAs and 5–8 μL QuickShuttel-Enhanced transfection reagent (Biodragon Immunotechnologies, Beijing, China) in Grace’s medium, respectively. And the cells were cultivated for 48 h at 27 °C. Finally, the cells were re-fed in a fresh Grace’s medium with 20E at a final concentration of 1 μM for 12 h. Equivalent volume of DMSO as control.

### The assay of larval feeding, body weight, and body length

The average quantity of feeding, body weight, and body length of insects from first instar larvae (1-F) to pupae at 2 day (P-2) were analyzed individually based 30 insects for three repeats after different treatments. The amount of diet actually eaten was estimated by the difference of the diet weight before and after testing twice every day, individually. The average weight and length of body were measured twice every day, individually. For the assay of the food consumption and increment body weight in 20E and flupentixol used the same method.

### Immunocytochemistry

HaEpi cells were washed three times with 500 μL DPBS, and fixed with 4% paraformaldehyde which diluted in PBS for 10 min in dark. The fixed cells were washed six times for 3 min each, and treated with 0.2% Triton-X 100 in PBS for 15 min. The cells were washed with DPBS six times for 3 min each, covered with blocking solution (2% bovine serum albumin (BSA) in PBS) for 1 h. The above buffer was exchanged with blocking solution contained antibodies against the target protein that was 1:100 diluted in 2% BSA/PBS for overnight at 4 °C. The cells were washed six times with DPBS for 3 min each and then the cells were treated with 200 μL goat anti-rabbit IgG Alexa Fluor 488 (Introvigen, Carlsbad, CA, USA) diluted with 1:1,000 for 1 h at 37 °C in dark. The cells washed 3 times with DPBS for 5 min each. Plasma membrane was stained with 200 μL Alexa Fluor 594-conjugated wheat germ agglutinin (WGA) (1:2,000 in PBS) (Introvigen, Carlsbad, CA, USA) in dark at room temperature for 10 min. Nuclei were stained with 4’, 6-diamidino-2-phenylindole (DAPI, 1 μg/mL in PBS) (Sigma, San Francisco, USA) in dark at room temperature for 10 min.

### Hematoxylin-eosin staining (HE) staining

The rehydrated histologic sections were stained with hematoxylin (1 g of hematoxylin, 10 mL of ethanol, 20 g of KAl(SO_4_)_2_, and 200 mL of H_2_O) for 10 min. Next, the sections were washed with running water for 1 min and stained with Scott’s liquid for 1 min. The sections were then washed with hydrochloric acid ethanol differentiation medium (70% ethanol in 1% hydrochloric acid) for 20 s and stained with Scott’s liquid for 1 min. Finally, sections were incubated with 0.5% water-soluble eosin dye solution for 30 s and subsequently washed with running water. The images were observed using an Olympus BX51 fluorescence microscope (Olympus Optical Co., Tokyo, Japan).

### Tunel assay

Tunel assay was conducted using the TUNEL BrightRed Apoptosis Detection Kit (Vazyme Biotech Co., Nanjing, China) according to the manufacturer’s instructions. The images were observed using an Olympus BX51 fluorescence microscope (Olympus Optical Co., Tokyo, Japan). The red fluorescence intensity (tunnel signal) was counted by ImageJ and the acquired data were analyzed by software of GraphPad.

### The detection of the intracellular Ca^2+^concentration

The cells were interfered by 2 μg dsRNAs or plasmids at 27 °C for 24 h after the density of cells up to 70–80%. Then the cells are treated with fresh medium included AM ester Calcium Crimson dye (Invitrogen, Carlsbad, CA, USA) (final concentration 3 μM) for 30 min at 27 °C. The cells were washed with Dulbecco’s PBS (DPBS; 137 mM NaCl, 2.7 mM KCl, 1.5 mM KH_2_PO_4_, and 8 mM Na_2_HPO_4_, pH 7.4) for three times. Then the cells were included with 20E in DPBS (final concentration 1 μM), and were automatic photographed once every 6 s for 2 min. After that, DPBS which including CaCl_2_ (final concentration 1 mM) and 20E (final concentration 1 μM), were put into microscope slides. Laser Scan Confocal Microscope Carl Zeiss LSM 700 (Thornwood, NY, USA) was used to detect the fluorescence at 555 nm every 6 s for 360 s. Finally, the fluorescence intensity of every collected photo was obtained by Image Pro-Plus software (Media Cybernetics, United States) and statistically analyzed.

### Intracellular cAMP assay

The HaEpi cells were cultured in 6-well plates to 80% density. dsRNAs were knocked down as above method. HaEpi cells were transfected with DopEcR-GFP, DopEcR-M-GFP, ErGPCR-2-GFP, and ErGPCR-2-M-GFP for 36 h. After 36 h, Grace’s medium was replaced with DPBS with 0.5 mM 3-isobutyl-1-methylxanthine (Sigma, St. Louis, MO USA). After 30 min, the cells were treated with 2 μM 20E for 0, 5, 10, 15, 30, 60, and 120 min; DMSO-treated cells served as a solvent control. The medium was removed and treated with 0.1 M HCl. The concentrations of cAMP in the cells were detected using the cAMP ELISA Kit (Biodragon, Beijing, China) according to the manufacturer’s instructions.

### Detection of cell proliferation and apoptosis

The HaEpi cells were treated with 5 μM 20E or 10 μM dopamine (DA) for 72 h. The 5-ethynyl-2′-deoxyuridine (EdU) kit (Ribobio, Guangzhou, China) was used to detect cell proliferation according to the manufacturer’s protocol. The NucView 488 caspase-3 assay kit (NO. 30029 Biotium, Hayward, USA) was used to detect the activity of caspase-3 in the HaEpi cells, according to the manufacturer’s instructions. The nuclei were stained with DAPI (10 μg/mL) for 10 min at room temperature in dark, and observed with Laser Scan confocal Microscope Carl Zeiss 700 (LSM 700) (Zeiss, Thornwood, ZY).

### Co-immunoprecipitation (Co-IP)

The ORFs of Gαs (GenBank accession no. MK134004), Gαq (GenBank accession no. AAX56092.1), and DopEcR were inserted into the pIEx-4-RFP-His or pIEx-4-His vectors, respectively. The reconstructed plasmids were then transfected into the HaEpi cells. The cells were incubated with 2 μM 20E for 30 min. DMSO was used as the control. The mouse monoclonal antibody against RFP (1 μL) and radioimmunoprecipitation assay (RIPA) buffer (0.1 M Tris-HCl buffer containing 150 mM NaCl, and 1% Nonidet P-40, pH 8.0) (400 μL) was incubated with protein A resin for 30 min at room temperature. The resin was washed with 500 μL RIPA buffer thrice. The protein was extracted from cells using RIPA buffer. The supernatant was harvested by centrifugation at 10,000 *g* for 10 min at 4 °C. The supernatant was added to protein A resin to eliminate nonspecific binding and harvested by centrifugation. The supernatant was added to the resin-antibody complex and incubated for 2–4 h with gentle shaking at 4 °C. The resin was harvested by centrifugation and washed with RIPA buffer thrice. The resin was treated with SDS-PAGE loading buffer and boiled for 10 min. After centrifugation at 12,000 *g* for 2 min (4 °C), the protein samples were detected by Western blotting with a primary antibodies of mouse monoclonal antibody against RFP and His (ABclonal, Wuhan, China). RFP-His was overexpressed in the HaEpi cells as a negative control as described above.

### Overexpression and phosphorylation analysis of CDK10, USP1, and PKAC1

The ORFs of CDK10 (GenBank accession no. KC188798), USP1 (GenBank accession no. EU526832), and PKAC1 (GenBank accession no. KT207930) were inserted into the pIEx-4-His vector. The reconstructed plasmids were transfected into the HaEpi cells for 36 h. The cells were then transfected with *dsDopEcR* or *dsGFP* for 24 h, followed by incubating with 2 μM 20E or DMSO for 30 min. DMSO was used as the control. The gel concentration of SDS-PAGE was 7.5%. 40 μL of protein (2 μg/μL) was incubated with 0.5 μL of λPP, 5 μL of buffer, and 5 μL of MnCl_2_ (50 μL total) at 30 °C for 30 min according to the manufacturer’s specifications (Millipore, Temecula, CA, USA). The sample was boiled for 10 min after adding the SDS-PAGE loading buffer and then detected by Western blot analysis.

### Chromatin immunoprecipitation (ChIP)

The ORF of EcRB1 (GenBank accession no. EU526831) was inserted into the pIEx-4-RFP-His vector. The pIEx-4-EcRB1-RFP-His plasmid was transfected into HaEpi cells with a 70% density in six-well plates for 48 h. Then, the cells were transfected with *dsDopEcR* or *dsGFP* for 24 h. Finally, the cells were treated with 20E or DMSO for 3 h. The cells were cross-linked with 1% formaldehyde at 37 °C for 10 min and then added glycine to final concentration of 0.125 M at 25 °C for 10 min to terminate the cross-linking. The cells were washed twice with PBS and then suspended with SDS lysis buffer (1% SDS, 10 mM EDTA, 50 mM Tris-HCl, pH 8.1). 200–1,000 bp DNA fragments were obtained by sonication. After centrifugation, supernatants were added to the Protein A resin and incubated at 4 °C for 1 h to pre-treat nonspecific binding. After centrifugation, one supernatant was used as an input sample for qRT-PCR. Other supernatants were incubated with anti-RFP antibody or mouse control IgG as a negative control at 4 °C overnight. Protein A resin was added into the immunoprecipitated protein-DNA complex and incubated at 4 °C for 2 h. The complexes were washed once with low-salt wash buffer (0.1% SDS, 1.0% Triton X-100, 2 mM EDTA, 200 mM Tris-HCl, pH 8.0, 150 mM NaCl), high-salt wash buffer (0.1% SDS, 1.0% Triton X-100, 2 mM EDTA, 20 mM Tris-HCl, pH 8.0, 500 mM NaCl), LiCl wash buffer (10 mM Tris-HCl, pH 8.1, 0.25 M LiCl, 1 mM EDTA, 1% NP-40, 1% deoxycholate) and twice with TE buffer (10 mM Tris-HCl, pH 8.1, 1 mM EDTA). The complexes were then washed with elution buffer (1% SDS, 0.1 M NaHCO_3_). The DNA-proteins were reversely cross-linked at 65 °C overnight, followed by RNase A and proteinase K treatments. The DNA was purified by phenol/chloroform extraction and analyzed by qRT-PCR using ChIP F/ChIP R primers (target to EcRE) (S2 Table).

### Structure modeling and ligand docking of GPCRs

The ligand 20E was docked into the active site of GPCRs using the Surflex-Dock (SFXC) function in the SYBYLx2.0 software (Tripos, St. Louis, MO, United States). Final figures were prepared with PyMOL program [59]. The molecular models of GPCRs binding steroid were created using the I-TASSER on-line server (https://zhanglab.ccmb.med.umich.edu/I-TASSER/).

### Overexpression of GPCRs in HaEpi cells and Sf9 cells

Proofreading DNA polymerase (TIANGEN, Beijing, China) was used to amplify the cDNA of GFP, ErGPCR-1 [8] (full length, GenBank number: JQ809653.1), ErGPCR-2 [9] (7TM region and added signal peptide, GenBank number: AKA95280.1), and *H*. *armigera* DopEcR (Gene Bank number: MG596302) using PCR with the primers (S2 Table). The resulting cDNAs were inserted into pIEx-4-GFP-His plasmid (pIEx-4 plasmid, Merck, Darmstadt, Germany, fused with GFP and a His-tag at the C-terminus by our laboratory). After the density of HaEpi cells to 70%–80%, the plasmid (5 μg/mL) was transfected into HaEpi cells using QuickShuttle-Enhanced transfection reagent (Biodragon Immunotechnologies, Beijing, China) in 2 mL Grace’s medium with 10% bovine serum at 27 °C for 48 h. Sf9 cells were utilized to expression GPCRs in insect cell culture medium ESF 921 (Expression Systems, Davis, California, USA) with 2% bovine serum because Sf9 expressed more exogenous protein than HaEpi cells.

### Site mutation of GPCRs

The GPCR binding sites were predicted online at http://zhanglab.ccmb.med.umich.edu/I-TASSER/ (S1 Table). GFP, GPCRs (ErGPCR-1, ErGPCR-2 and DopEcR) and their mutants [ErGPCR-2-M: Serine 113 to alanine (S113A), Cysteine 138 to alanine (C138A), Glycine 142 to alanine (G142A); DopEcR-M: Tyrosine 68 to alanine (Y68A), Tyrosine 109 to alanine (Y109A), Threonine 113 to alanine (T113A), Tryptophan 160 to alanine (W160A)] were amplified via bridged PCR method with the site mutated primers (The primers were based on the sequence around the mutated site, which were not showed because too many of the primers were used). The mutation was confirmed by DNA sequence.

### Detection of the 20E quantity bound by the cell membranes of HaEpi cells

DopEcR-GFP, ErGPCR-1-GFP, ErGPCR-2-GFP and their mutants, ErGPCR-2-M-GFP and DopEcR-M-GFP were overexpressed in HaEpi cells in a 25-cm^2^ cell culture bottle, respectively. The cells were washed with DPBS for 2 min twice. The cells were incubated in Grace’s medium containing 1 μM 20E for 5 min at 27 °C to allow 20E to bind to the cell membrane. Cells were collected by centrifugation at 1,700 × *g* and 4 °C for 5 min and the pellet was resuspended in 500 μL enzyme immunoassay (EIA) buffer (Bertin Pharma, Paris, France) and sonicated for 5 min. After centrifugation at 48,000 × *g* and 4°C for 1 h, the pelleted cell membrane debris was resuspended in 100 μL EIA buffer. 50 μg cell membrane proteins with fixed 20E in 50 μL EIA buffer was added with 450 μL EIA buffer and used to quantify 20E. A 20-Hydroxyecdysone Enzyme Immunoassay kit (20E-EIA kit) (Bertin Pharma, Paris, France) was used to detect cell membrane bound-20E according to the manufacturer’s instructions.

### Isolation of GPCRs and detection of the bound 20E

GPCRs were overexpressed in Sf9 cells in a 25-cm^2^ cell culture bottle using pIEx-4-GFP-His plasmid. After 48 h, total plasma membrane proteins were extracted using the kit of the cell transmembrane protein extracts (BestBio, Shanghai, China) and GFP were extracted using the RIPA Lysis Buffer (20 mM Tris, pH 7.5, 150 mM NaCl, 1% Triton X-100) without EDTA (Ethylenediaminetetraacetic acid) (Beyotime, Shanghai, China). 100 μL slurry of Ni^2+^ resin was washed three times with charge buffer (50 mM NiSO_4_) and once with binding buffer (0.5 M NaCl, 20 mM Tris-HCl, pH 7.9, 5 mM imidazole) for 5 min. The overexpressed GPCRs were bound on the washed Ni^2+^ resin (GE Healthcare, Pittsburgh, USA). Each 100 μL slurry of Ni^2+^ resin was added to 150 μg/50 μL of the plasma membrane proteins with 1 mL binding buffer on a four-dimensional rotating mixer for 40 min at 26 °C. Then, the Ni^2+^ resin was washed three times with binding buffer followed by three times with wash buffer (1 M NaCl, 20 mM Tris-HCl, pH 7.9), each for 5 min. After centrifugation at 500 × *g* for 1 min at 4 °C, the GPCR-resin were incubated with 1 μM 20E in 1 mL binding buffer for 5 min. The GPCR and bound 20E were washed three times with wash buffer, each for 5 min. The GPCR and fixed 20E were eluted using 50 μL elute buffer (0.5 M NaCl, 20 mM Tris-HCl, pH 7.9, 75 mM imidazole) and then diafiltration were carried out three times with EIA buffer using Amicon Ultra-15 (Merck Millipore, Temecula, California, United States of America) to reduce the concentration of imidazole for the following experiment. The concentration of the isolated GPCR was detected as 1˗1.5 μg/μL using the Bradford method. The 20E bound by 5–10 μg/5–7 μL eluted GPCR was added EIA buffer to 50 μL and detected using a 20E EIA kit as above method.

### 20-hydroxyecdysone enzyme immunoassay

The 20-hydroxyecdysone enzyme immunoassay (20E-EIA) is based on competition between unlabeled 20E (free 20E) and acetylcholinesterase (AChE)-labelled 20E (Tracer) for limited-specific rabbit anti-20E antiserum. The rabbit anti-20E antiserum was bound to a mouse monoclonal anti-rabbit antibody coated-plate. The plate was then washed using the wash buffer provided with the kit (Dilute 2 mL of concentrated Wash Buffer #A17000 with 800 mL of UltraPure water. Add 400 μL of Tween20 #A12000) to remove any unbound reagent. The AChE-labelled 20E and free 20E in samples were added into the wells and the plates were incubated at 4 °C overnight. After washing three times with wash buffer, Ellman’s reagent (an enzymatic substrate for AChE and chromogen) was added to the wells and incubated for 1.5 h at room temperature on a shaker. The AChE-labelled 20E acts on substrate in the Ellman’s Reagent to form a yellow compound that strongly absorbs light at 414 nm. The intensity of the color was determined at 414 nm by spectrophotometry (Infinite M200PRO NanoQuant, Tecan, Grödig, Austria); the optical density was proportional to the amount of tracer bound to the well and inversely proportional to the amount of 20E in the samples. The quantity of 20E bound to GPCRs was determined using a 20E standard curve generated using the same method. A detail protocol is provided with the kit.

### Saturation binding curve of GPCRs to 20E

GPCRs and GFP were overexpressed in Sf9 cells and isolated as above description. Proteins were stored at -80°C for saturation experiments. 20E-EIA was used for binding saturation curve analysis. GPCR bound on Ni^2+^ resin was incubated with 1 to 60 nM 20E in 250 μL binding buffer (20 mM HEPES, 100 mM NaCl, 6 mM MgCl_2_, 1 mM EDTA, and 1 mM EGTA) at 26 °C for 40 min. The GPCR and 20E bound resin was washed three times with wash buffer, each for 5 min and finally eluted in 50 μL elute buffer (0.5 M NaCl, 20 mM Tris-HCl, pH 7.9, 75 mM imidazole). The diafiltration of eluted protein was carried out with EIA buffer using Amicon Ultra-15 for three times. Protein concentration was detected by BCA method. 10 μg GPCR and its bound 20E was added EIA buffer to 50 μL and detected using a 20E EIA kit as above method. Nonspecific binding was determined in 20E-EIA assays with GFP. Dissociation constants (Kd) was determined by nonlinear regression from specific and nonspecific binding data of saturation experiments by using GraphPad Prism 7 on the assumption that 20E binds to a single site.

### Saturation binding curve of DopEcR to DA and the assay of 20E displacement

ELISA (Enzyme Linked Immunosorbent Assay, ELISA) plates were incubated with 10 μg of DopEcR-GFP isolated from HaEpi cells that were overexpressed DopEcR-GFP in 200 μL coating buffer (0.015 M Na_2_CO_3_, 0.035 M NaHCO_3_, pH 9.6) at 4 °C overnight. The plates were washed 3 times with wash buffer (0.15 M PBS, pH 7.4) and then incubated with 1% BSA in 200 μL PBS at 37 °C for 1 h. The plates were washed 3 times with wash buffer and added various concentrations of DA in 200 μL PBS. Remaining experiments were performed according to the manufacturer’s instructions of insect hemolymphal dopamine ELISA kit (MLBIO Biotechnology, Shanghai, China). The ELISA plates coated with DopEcR-GFP were incubated with 20 pmol of DA, and the increasing concentrations of the different ligands, 20E or DA, were incubated with the plates, respectively, to study the competition of 20E to DA.

### Statistical analysis

SPSS 23.0 (SPSS Inc., Chicago, IL, USA) was used for data analysis. All data come from at least three biologically independent experiments. Two-group datasets were analyzed by Student’s *t*-test. One-way analysis of variance (ANOVA) were used to analyzed different among three or more group by Duncan’s multiple comparison test was used at *p* = 0.05. One asterisk was used for *p* < 0.05, two asterisks for *p* < 0.01. The detail was showed in the related figure legends.

## Supporting information

S1 Fig DopEcR located in plasma membrane without internalization by 20E induction in HaEpi cells. 20E treatment (1 μM). DMSO as solvent control. **Green**: DopEcR protein stained with an anti-DopEcR antibody and secondary antibody labeled with Alexa-488. **Blue**: nucleus stained with 4’-6-diamidino-2-phenylindole dihydrochloride (DAPI). Observed by confocal microscope. Scale bar = 25 μm.
(TIF)

S2 Fig Modeled structures of GPCRs and the ligand. **CLR**, cholesterol, cholest-5-en-3beta-ol, cholesterin. **Y01**, chosterol hydrogen succinate, chosterol hemissuccinate, chosterol hemisuccinate, chosterol succinate, succinic acid monocholesterolester (modeling by http://zhanglab.ccmb.med.umich.edu/I-TASSER/).
(TIF)

S3 Fig Phylogenetic tree of DopEcR in various species. *The octopamine receptor Oamb isoform X1 (XP_021194395.1) in this article was named “DopEcR” in the previous article [10].
(TIF)

S1 Table Predicted binding residues and point mutations. (DOCX)

S2 Table The PCR primer sequences used in this experiment. (DOCX)
