# Supplementary figures and images for: The steroid hormone 20-hydroxyecdysone binds to dopamine receptor to repress lepidopteran insect feeding and promote pupation
Source: PLoS Genet. 2019 Aug 14;15(8):e1008331. doi: 10.1371/journal.pgen.1008331 (PMC6693746; doi:10.1371/journal.pgen.1008331)

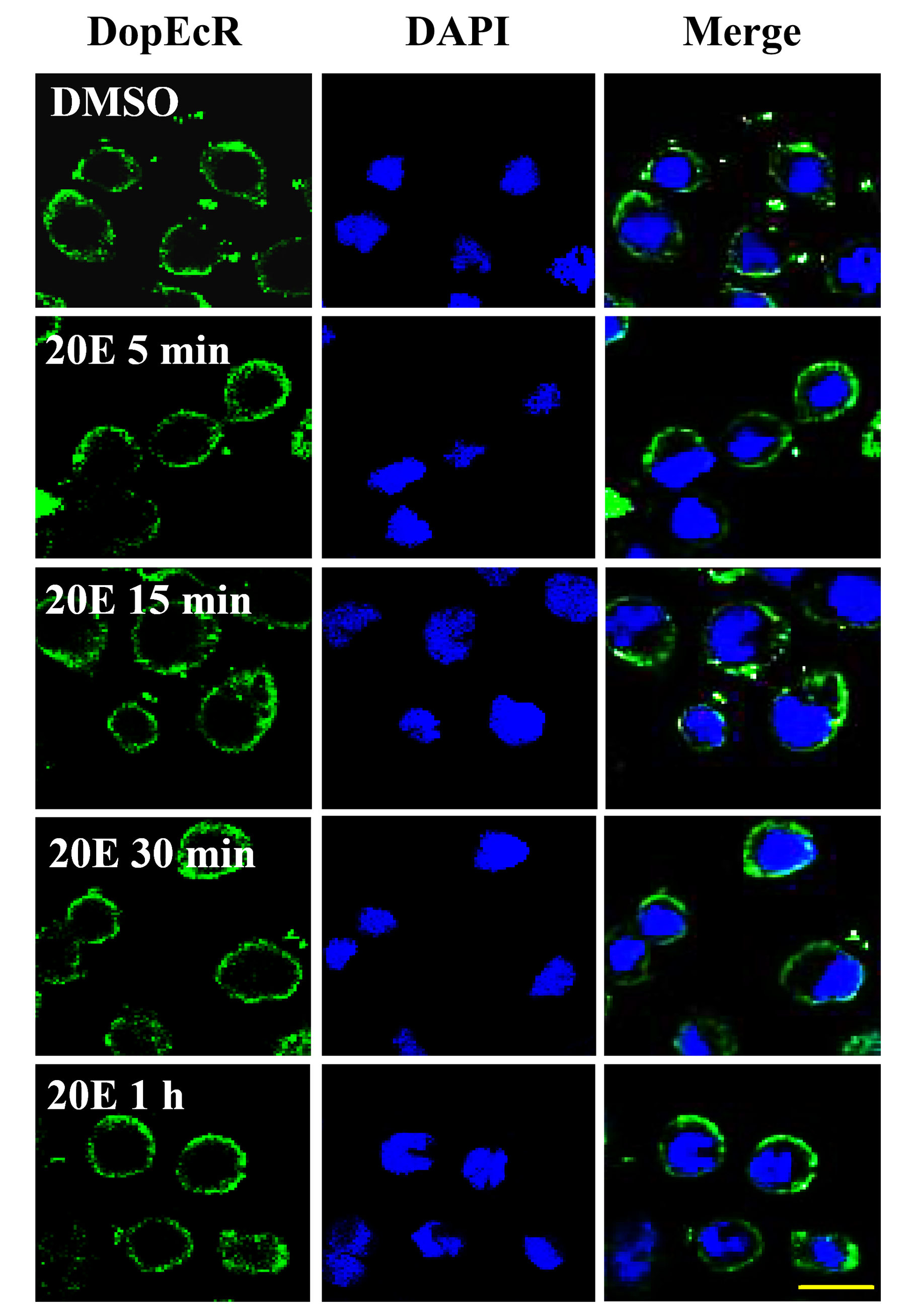

Supplement: S1 Fig — 20E treatment (1 μM). DMSO as solvent control. Green: DopEcR protein stained with an anti-DopEcR antibody and secondary antibody labeled with Alexa-488. Blue: nucleus stained with 4’-6-diamidino-2-phenylindole dihydrochloride (DAPI). Observed by confocal microscope. Scale bar = 25 μm. (TIF) [file pgen.1008331.s001.tif]

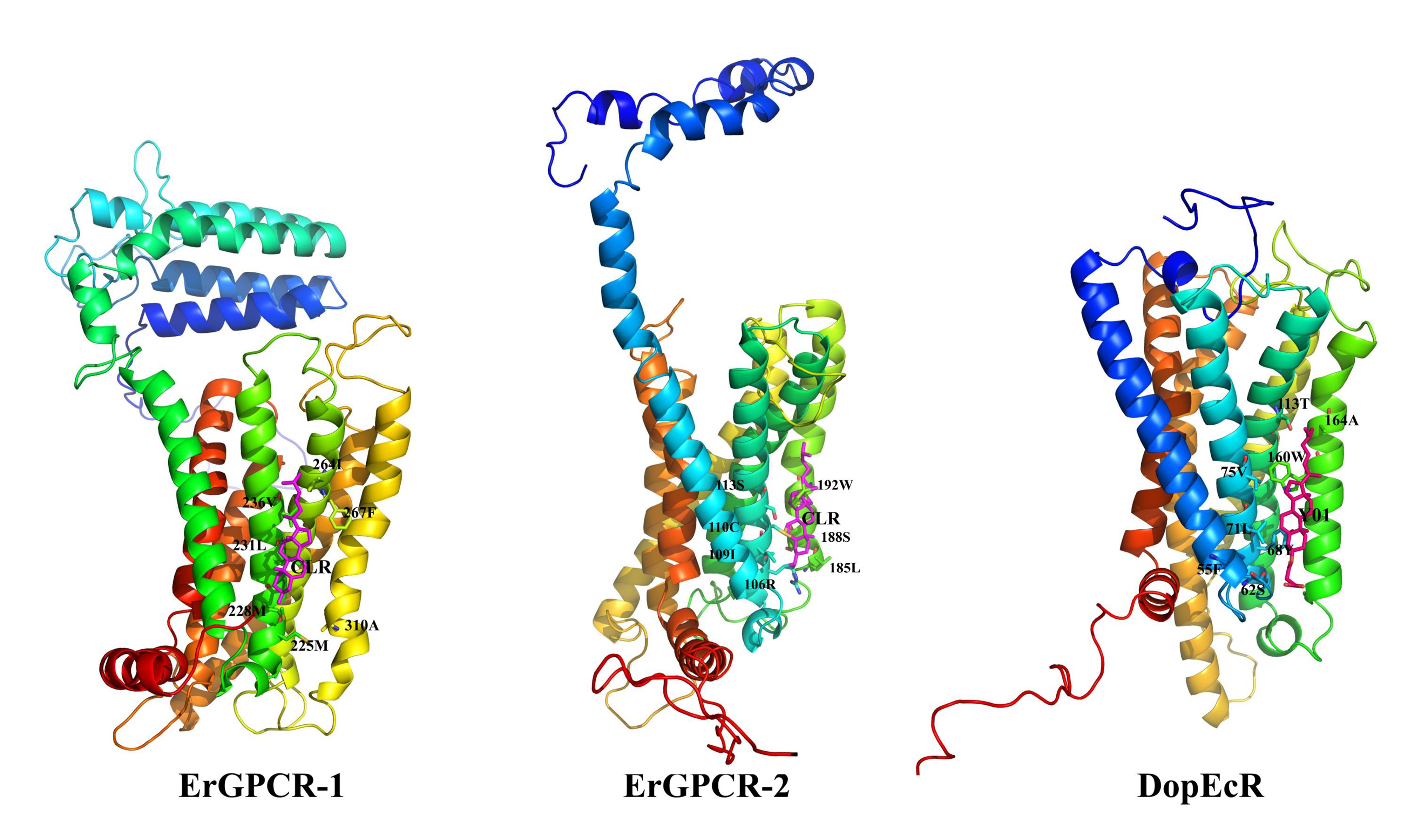

Supplement: S2 Fig — CLR, cholesterol, cholest-5-en-3beta-ol, cholesterin. Y01, chosterol hydrogen succinate, chosterol hemissuccinate, chosterol hemisuccinate, chosterol succinate, succinic acid monocholesterolester (modeling by http://zhanglab.ccmb.med.umich.edu/I-TASSER/). (TIF) [file pgen.1008331.s002.tif]

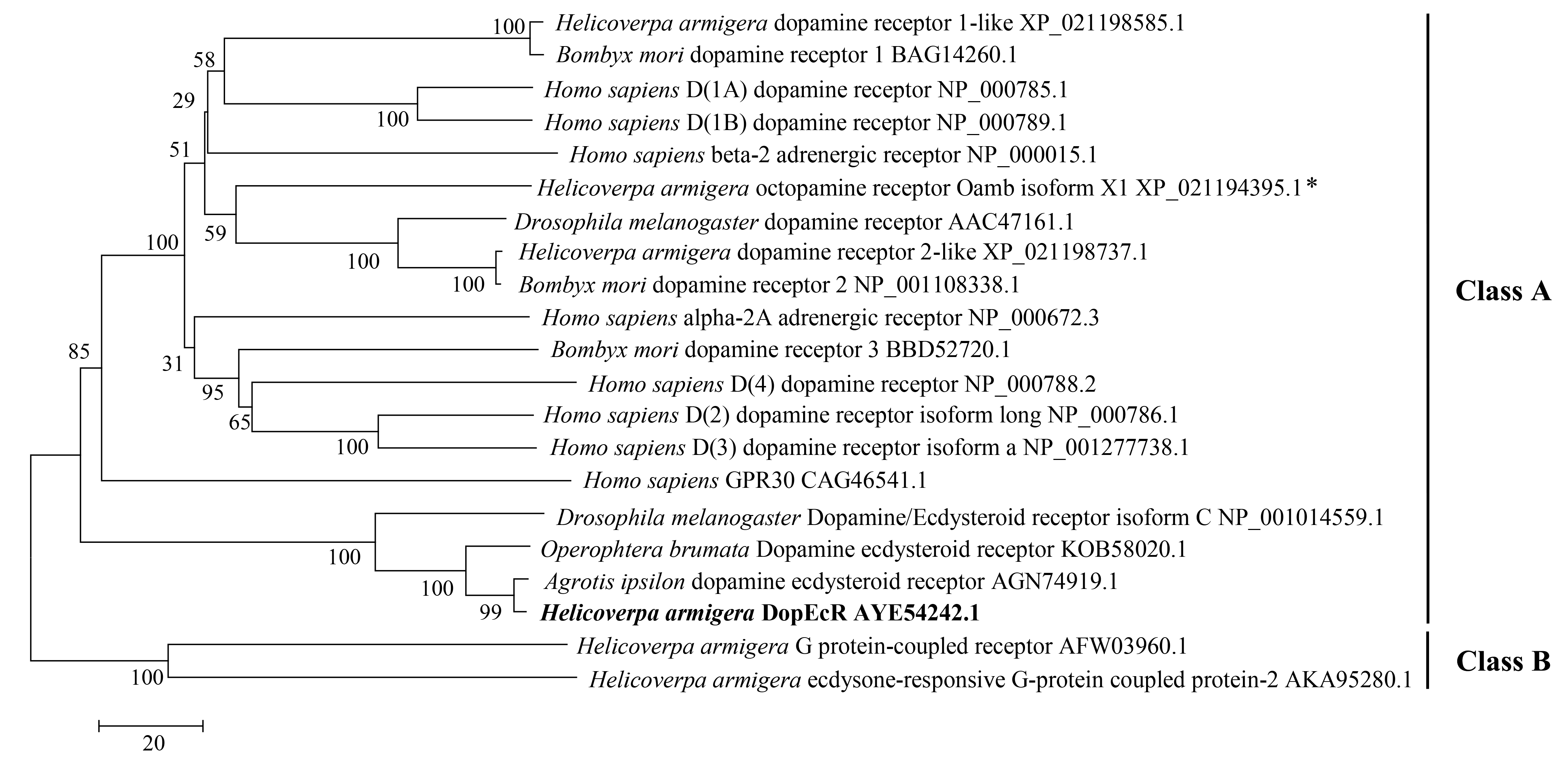

Supplement: S3 Fig — *The octopamine receptor Oamb isoform X1 (XP_021194395.1) in this article was named “DopEcR” in the previous article [10]. (TIF) [file pgen.1008331.s003.tif]
