# Supplementary material for: The steroid hormone 20-hydroxyecdysone binds to dopamine receptor to repress lepidopteran insect feeding and promote pupation
Source: PLoS Genet. 2019 Aug 14;15(8):e1008331. doi: 10.1371/journal.pgen.1008331 (PMC6693746; doi:10.1371/journal.pgen.1008331)
Supplement: S1 Table — (DOCX) [file pgen.1008331.s004.docx]

**Table S1.** **Predicted binding residues and point mutations.**

| **Proteins** | **C-score** | **Ligands** | **Binding sites** | **Mutation sites** |
| --- | --- | --- | --- | --- |
| ErGPCR-1  ErGPCR-2  DopEcR | 0.03  0.05  0.10 | CLR  CLR  Y01 | 225M, 228M, 232L, 236V, 264I, 267F, 310A  106R, 109I,110C, 113S, 138C, 142G, 185L,188S,192W  55F, 62S, 68Y, 71L, 75V, 109Y, 113T, 160W, 164A | S113A, C138A, G142A  Y68A, Y109A, T113A, W160A |

The predicted binding residues of ErGPCR-1, ErGPCR-2, and DopEcR are predicted online at http://zhanglab.ccmb.med.umich.edu/I-TASSER/. **C-score** is the confidence score of the predicted binding site. **CLR,** cholesterol, cholest-5-en-3beta-ol**,** cholesterin. **Y01**, chosterol hydrogen succinate, chosterol hemissuccinate**,** chosterol hemisuccinate, chosterol succinate, succinic acid monocholesterolester. ErGPCR-2 was numbered from the overexpressed 7TM.
