# Supplementary material for: The steroid hormone 20-hydroxyecdysone binds to dopamine receptor to repress lepidopteran insect feeding and promote pupation
Source: PLoS Genet. 2019 Aug 14;15(8):e1008331. doi: 10.1371/journal.pgen.1008331 (PMC6693746; doi:10.1371/journal.pgen.1008331)
Supplement: S2 Table — (DOCX) [file pgen.1008331.s005.docx]

**Table S1. The PCR primer sequences used in this experiment**

| Primer name | (5'→3') nucleotide sequence |
| --- | --- |
| **RNAi primers** |  |
| DopEcR-RNAiF | gcgtaatacgactcactataggggtggtgccgctgtccgtat |
| DopEcR-RNAiR  DopEcR-swRNAiF  DopEcR-swRNAiR  GFP-RNAiF  GFP-RNAiR  GFP-swRNAiF  GFP-swRNAiR | gcgtaatacgactcactataggttccgtgtcgaaattagcttc  tactcaagatctggtggtgccgctgtccgtat  tactcaggtaccttccgtgtcgaaattagcttc  gcgtaatacgactcactataggtggtcccaattctcgtggaac  gcgtaatacgactcactataggcttgaagttgaccttgatgcc  tactcagcggccgctggtcccaattctcgtggaac  tactcactcgagcttgaagttgaccttgatgcc |
| **qRT-PCR primers** |  |
| DopEcR-QF | actacgtatgcttacacggcca |
| DopEcR-QR | tgagccagaacgtgcagaccat |
| HHR3-QRTR1 | gactttgctgatgtcaccctccgc |
| HHR3-QRTF1  EcRB1-QRTF1  EcRB1-QRTR1  USP1-QRTF1  USP1-QRTR1  BrZ7F  BrZ7R  RpL27F  RpL27R | tcaagcacctcaacagcagcccta  aattgcccgtcagtacga  tgagcttctcattgagga  ggtcctgacagcaatgtt  ttccagctccagctgactgaag  ggtgactgtccttactgcggc  ttaattcctttgaccatgact  acaggtatccccgcaaagtgc  gtccttggcgctgaacttctc |
| β-actinF | cctggtattctgaccgtatgc |
| β-actinR  CHIP F  CHIP R | ctgttggaaggtggagagggaa  atattcgaatcgttggcg  cagtgtaattaagagaca |
| **Expression**  HaDopECR-expF  HaDopECR-expR  **Overexpression** | tactcagaattacatcattgcctcgtttctca  tactcactcgagctcgggttggacaggttct |
| OV ErGPCR2-F* | tactcagagctcatgattacattcataacagtg |
| OV ErGPCR2-R | tactcactgcagaggctgtttgatgttgagcgac |
| OV ErGPCR1-F | tactcactgcagatgattacattcataaca |
| OV ErGPCR1-R | tactcaggtaccaaattcgccattagtcgt |
| OV DopEcR-F | tactcagagctcatgcctgccaagatgatgagc |
| OV DopEcR-R  OV Gαq-F  OV Gαq-R  OV Gαs-F  OV Gαs-R | tactcaagatctggtcgtcgttgtccaggccgat  tactcagagctcatggagtgctgcatgtcgg  tactcactgcagtgctagattaaattccttga  tactcagagctcatgggatgcttcggctcg  tactcaagatctggagcagctcgtactgccg |

^*^

* The signal peptide from ErGPCR-1 was added in the front of 7TM (amino acid 359–757) of ErGPCR-2 to localize the 7TM of ErGPCR-2 on the cell membrane.
